# Cognitive Radio–Based Ionospheric Scintillation Detection: A Low-Cost Framework for GNSS Detection and Monitoring in Equatorial Regions

**DOI:** 10.3390/s26061765

**Published:** 2026-03-11

**Authors:** Jaime Orduy Rodríguez, Walter Abrahao Dos Santos, Claudia Nicoli Candido, Danny Stevens Traslaviña, Cristian Lozano Tafur, Pedro Melo Daza, Iván Felipe Rodríguez Barón

**Affiliations:** 1 Instituto Nacional de Pesquisas Espaciais, Sao Jose dos Campos 12227-010, SP, Brazil; walter.abrahao@inpe.br (W.A.D.S.); claudia.candido@inpe.br (C.N.C.); 2 Faculty of Engineering and Basic Sciences, Fundación Universitaria los Libertadores, Main Campus, Bogota 111221440, Colombia; dstraslavinan@libertadores.edu.co (D.S.T.); clozanot@libertadores.edu.co (C.L.T.); pfmelod@libertadores.edu.co (P.M.D.); ifrodriguezb@libertadores.edu.co (I.F.R.B.); 3 Centro de Educación Militar, Escuela de Aviación del Ejército, Bogota 110911, Colombia

**Keywords:** ionospheric scintillation, cognitive radio, GNSS, space weather monitoring, machine learning

## Abstract

**Highlights:**

**What are the main findings?**

- The proposed Low-Cost Scintillation Laboratory (LCSL), integrating Software-Defined Radio, Cognitive Radio, and Machine Learning, demonstrated effective real-time detection and monitoring of ionospheric scintillation in equatorial and low-latitude regions, with successful validation using data from Bogotá, Cartagena, and Santa Marta.
- The comparative evaluation of cognitive radio algorithms showed that DIMSUMnet provides superior GNSS signal stability, higher C/N_0_, and faster interference detection under scintillation conditions, outperforming Adapt4 XG1 in environments affected by equatorial plasma bubbles.

**What are the implications of the main findings?**

- The results confirm that low-cost, cognitive radio–based infrastructures can significantly enhance GNSS resilience in countries with limited space weather monitoring capabilities, reducing dependence on expensive commercial scintillation receivers.
- The demonstrated adaptability of cognitive radio and ML-based approaches supports their use as a scalable foundation for national and regional ionospheric monitoring networks, with direct benefits for aviation, navigation, and satellite-dependent critical systems in equatorial regions.

**Abstract:**

Global Navigation Satellite Systems (GNSS) are highly affected in equatorial regions, especially due to the formation of Equatorial Plasma Bubbles (EPBs), which cause disturbances in the ionosphere resulting in different forms of signal degradation. Despite Colombia’s privileged geographic position, its limited monitoring infrastructure hinders the detection and mitigation of these effects. This study proposes the development of a Low-Cost Scintillation Laboratory (LCSL) using a cognitive radio–based approach for real-time scintillation monitoring, aimed at improving GNSS reliability. The system was designed following a Systems Engineering methodology, defining functional architectures and constraints. A communication system model was developed to account for EPBs’ effects on GNSS signals, while cognitive radio algorithms within a Software-Defined Radio (SDR) framework enabled real-time detection, monitoring, and alert generation. To implement this approach, monitoring stations were deployed in Bogotá, Cartagena, and Santa Marta utilized low-cost GNSS receivers integrated with Machine Learning (ML) algorithms for the automatic classification of scintillation events. Additionally, the system’s accuracy was validated by comparing experimental data with historical records from the Geophysical Institute of Peru (IGP). The results demonstrated that the integration of cognitive radio and ML-based detection enhanced precision and adaptability compared to traditional methods. The network of monitoring stations effectively validated the system’s performance, providing valuable insights into equatorial ionospheric dynamics. This study contributes to the advancement of monitoring methodologies and highlights the importance of accessible infrastructure for mitigating EPB effects on GNSS, ultimately fostering more resilient navigation and communication systems.

## 1. Introduction

The ionosphere consists of a mixture of free electrons and positive ions, generally present in equal numbers, forming a partially ionized gas that can be described as plasma. It constitutes the upper region of the Earth’s atmosphere, where ionization occurs primarily through the interaction of solar radiation with neutral atmospheric constituents, mainly atomic oxygen [1,2,3]. The energy required for this process is provided by the Sun in the form of extreme ultraviolet (EUV) and X-ray radiation, which ionize atoms and molecules in the neutral atmosphere through a mechanism known as photoionization [1,4].

This atmospheric layer extends approximately from 60 km to 1000 km in altitude and is conventionally divided into three main regions: the D-layer, located between about 50 km and 90 km; the E-layer, extending from 90 km to 120 km; and the F layer, which ranges from approximately 120 km up to 1000 km [5].

Nevertheless, the ionosphere exhibits complex and highly dynamic geophysical characteristics, which become particularly pronounced in low-latitude regions, especially near the magnetic equator. In these regions, the nearly horizontal orientation of the geomagnetic field, combined with its perpendicularity to both the meridional neutral wind and the background electric field, gives rise to distinctive electrodynamic processes that promote the development of plasma irregularities. Furthermore, the high levels of solar ionizing radiation at low latitudes enhance the intensity and variability of these phenomena [6,7,8].

Among the most significant ionospheric phenomena in equatorial regions are the Equatorial Ionization Anomaly (EIA), the Equatorial Electrojet (EEJ), and plasma irregularities, all of which have a substantial impact on technologies that depend on ionospheric propagation conditions, such as radio communication and satellite-based navigation systems [5,9]. These phenomena predominantly occur in the E and F layer [9,10].

Within this context, the most critical phenomena are the temporal and spatial irregularities in the density of the ionospheric plasma, which manifest as depletions in Total Electron Content (TEC) [7,11]. When a radio signal propagates through the ionosphere, such irregularities induce diffraction and scattering effects, leading to random fluctuation and rapid changes in signal amplitude and phase over short periods. These disturbances are called scintillation [12].

These disturbances are classified into amplitude ionospheric scintillation (amplitude scintillation) and phase ionospheric scintillation (phase scintillation) [6]. Their severity depends on ionospheric variability, which, in turn, is influenced by factors such as the time of day, the Sun’s position, and its radiation. Additionally, their occurrence follows a temporal pattern, varying on daily, seasonal, and yearly scales. At low latitudes, scintillation is more prevalent during the vernal and autumnal equinoxes due to enhanced ionospheric instability, while at high latitudes, it is closely linked to geomagnetic activity, which tends to peak during winter months [2,5,13,14].

Ionospheric scintillation is categorized based on the magnetic latitude at which it occurs. Low-latitude scintillation occurs at approximately ±20° magnetic latitude; middle-latitude scintillation is observed between 20° and 60°; and high-latitude scintillations, particularly in polar areas, occur at latitudes above 60° [15,16].

Low-latitude scintillation, particularly in equatorial regions, is the most intense and frequent due to equatorial plasma irregularities, such as EPBs, which develop around sunset and can significantly disrupt radio signals. Here, the amplitude scintillation is more intense than in high-latitude scintillation [14,16].

Middle-latitude scintillation is generally less intense than that observed in low and high altitudes. The disturbances in these regions often result from the extension of the phenomena occurring at high and low latitudes. The intrinsic variability of EPBs, linked to their spatial extension, can lead to the occurrence of scintillation even under quiet geomagnetic conditions. Although less intense than in equatorial and polar regions, its occurrence can still affect communication and navigation systems [8,17].

High-latitude scintillations, while also significant, are primarily driven by magnetic storms and auroral activity. These events create strong ionospheric irregularities, leading predominantly to phase scintillation, which affects Global Positioning System (GPS) signals and other radio communication systems. At these latitudes, phase scintillation is more frequent and intense than in equatorial regions [14,16].

Although scintillation is observed across all three major global regions, this phenomenon is most intense and unpredictable in equatorial and low-latitude regions [18], where its occurrence is primarily associated with the formation of EPBs.

The post-sunset enhancement of upward plasma drift plays a crucial role in the generation of EPBs and, consequently, Equatorial Spread-F (ESF). During the day, the eastward electric field generated in the ionospheric E-region induces a vertical E × B drift, lifting the F-region plasma at the equator. However, as solar ionization in the E-region decreases in the late afternoon, the ion density declines, reducing the E-region’s conductivity and allowing the F-region dynamo to become the dominant driver of ionospheric electrodynamics [19,20].

This transition, combined with the sharp conductivity gradient across the solar terminator, leads to an enhancement of the eastward electric field after sunset, known as the Pre-Reversal Enhancement (PRE). This intensified electric field further strengthens the vertical plasma drift, elevating plasma to altitudes exceeding 500 km [19,20].

At these higher altitudes, recombination rates are lower, allowing regions of enhanced plasma density to persist beyond local midnight, thereby reinforcing the equatorial anomaly. Meanwhile, the rapid decay of the lower ionosphere after sunset creates a steep plasma density gradient at the base of the uplifted F-region. This gradient establishes the necessary conditions for the Rayleigh-Taylor instability, which can be further amplified by external perturbations, such as atmospheric gravity waves [11]. The growth of this instability results in the formation of plasma bubbles-localized regions of depleted plasma density responsible for ESF [19,20].

**Figure 1:**
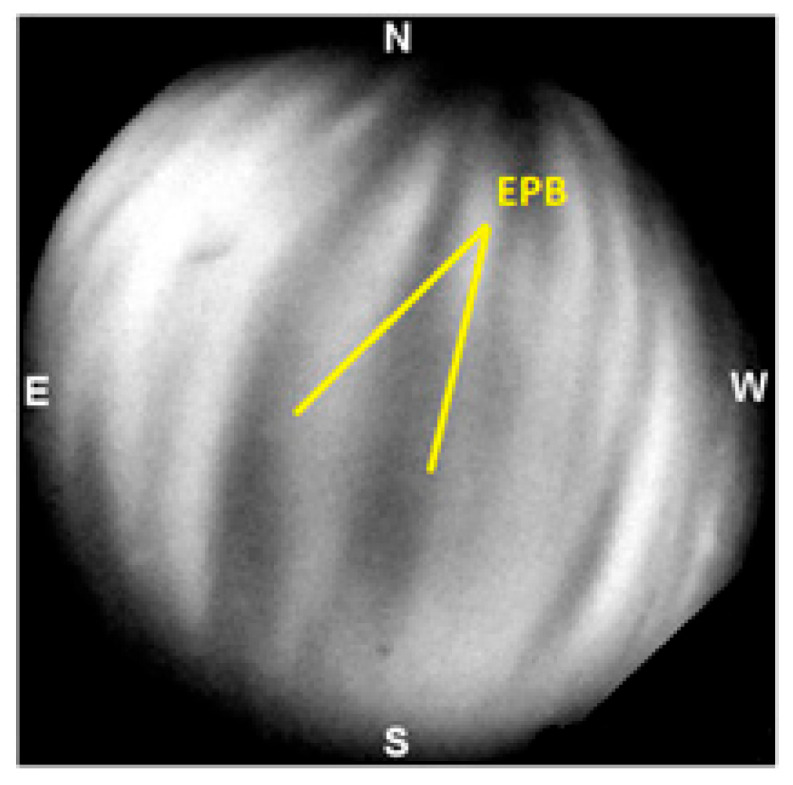
All-sky airglow image showing the development of an Equatorial Plasma Bubble (EPB). The yellow arrows indicate the apparent propagation direction of the EPB structures. Cardinal directions (N, S, E, W) are indicated for reference, and the Observation time was 01:38:08 UTC-3, adapted from [19].

As these plasma bubbles ascend, they generate small-scale plasma irregularities that disrupt the smooth propagation of radio waves. An example of EPBs can be seen in Figure 1. These irregularities induce strong fluctuations in signals from Very High Frequency (VHF) to L-band and can also cause disruptions in signals emitted by both low and high-altitude orbiting satellites, severely impacting telecommunications and satellite-based navigation systems [18,19,20]. The accuracy and integrity of received signals are sometimes severely affected to the point of complete loss. Among the most affected systems are those used for navigation and satellite communication, such as GNSS and SATCOM.

These disturbances can cause significant disruptions, leading to phase errors associated with carrier signal fading, increased instability in Doppler shift measurements, and, in severe cases, complete signal loss. Such effects may result in positioning inaccuracies of several meters or even total communication outages [11,15]. Navigation systems such as the GPS support a wide range of applications, including en-route, terminal, and other non-precision operations, particularly in safety-critical domains such as aviation, where high positioning accuracy and signal continuity are essential.

**Figure 2:**
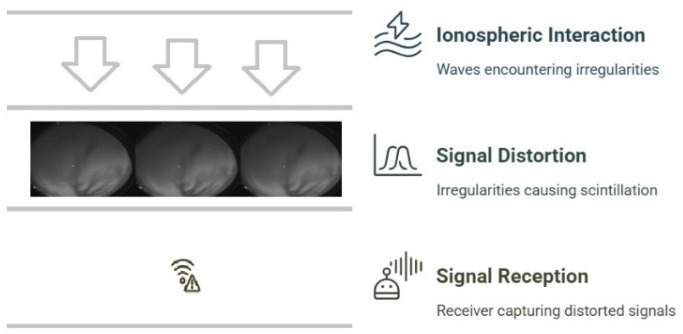
Conceptual representation of the interaction between GNSS signals and ionospheric plasma irregularities. First, GNSS signal propagation occurs through ionospheric irregularities. Second, plasma density depletions distort the signal, producing amplitude and phase scintillation. Third, the distorted signal is received by a ground-based GNSS receiver, where scintillation indices are measured. Adapted from: [23].

As the aviation industry continues to grow, the supporting navigation infrastructure must become increasingly sophisticated. However, despite the implementation of various techniques aimed at enhancing GNSS resilience, these systems remain vulnerable to space weather phenomena, including solar flares and geomagnetic storms [21,22]. Figure 2 illustrates the signal distortion process within the ionosphere, from wave interaction with plasma irregularities to the reception of the degraded signal at the receiver.

These vulnerabilities in navigation and communication systems highlight the need for continuous ionospheric monitoring to mitigate their effects on critical applications such as aviation, defense, and geospatial mapping [5].

### 1.1. EPBs Occurrence Features

Shetti et al. [24] present the monthly, seasonal, and annual variation in the ionospheric TEC and the percentage of occurrence rate of EPBs during the lowest to highest solar activity phase for the period of 2002–2013, the results point to all the seasons TEC obey solar activity, however it is seen that the TEC in vernal equinox is marginally larger during the period 2002–2007 while autumnal TEC is maximum during 2008–2013. This shows that the seasonal TEC is not only dependent on the solar cycle but also on the phases of the solar cycle. They concluded that the percentage of occurrence of EPBs is positively correlated with solar activity. And during both disturbed and quiet periods, the percentage of plasma bubbles is positively correlated with solar activity; however, disturbed nights suppress the EPBs occurrence.

**Figure 3:**
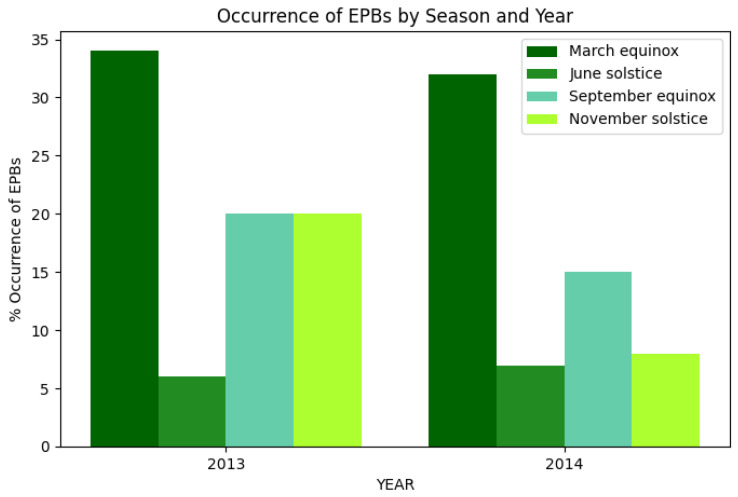
Seasonal occurrence of EPBs over Kenya during 2013–2014. The bar chart shows the percentage of EPB occurrences observed during the March equinox, June solstice, September equinox, and November solstice for each year [25].

Subsequently, ref. [25] presents a study related to the occurrence characteristics of EPBs over Kisumu, Kenya during Solar maximum of Solar Cycle 24, in this report it was described the seasonal occurrences of EPBs for the years 2013 and 2014, they classified the results over the four seasons for each year where the March equinox comprised of months of March and April; June solstice comprised of months of June and July; September equinox comprised of months of August and September while the November solstice compromised of months of November and December, as can see in Figure 3.

The conclusion was that the major percentage of EPBs occurrence is on March equinox with a 33.33% for 2013 and 30.76% for 2014, this behavior according to Nyongesa et al. [25] is because during equinoctial period, the solar terminator is aligned with the local geomagnetic field lines and hence there is increased photoionization resulting from high SEUV radiation during the period, leading to formation of irregularities [25]. Huang et al. [26] have shown that the percentage of EPBs occurrence increases during periods of high solar activity.

### 1.2. Instrumentation for Measuring Ionospheric Scintillation EPBs

Ionospheric scintillation, caused by plasma irregularities such as EPBs, disrupts the propagation of Radio Frequency (RF) signals, affecting satellite-based navigation, telecommunications, and remote sensing applications. To monitor and mitigate these disturbances, various ground-based and space-based instruments have been developed, each providing complementary data on different aspects of ionospheric dynamics. Among the most widely used instruments are GNSS scintillation receivers, which measure amplitude (S_4_ index) and phase (σφ) scintillation, allowing for real-time tracking of ionospheric disturbances. These receivers, such as the Septentrio PolaRx5S, have proven to be essential tools for high-precision monitoring [27]. Another crucial technology is the ionosonde, which operates by transmitting high frequency (HF) radio pulses into the ionosphere to determine electron density profiles and ionospheric layer heights. Advanced systems like the DPS-4D and AIS-INGV provide valuable data for understanding plasma irregularities associated with scintillation [28].

Beyond traditional ionospheric sounders, other instruments contribute significantly to scintillation research. Coherent beacon systems, such as the Coherent Electromagnetic Radio Tomography (CERTO) system, enable tri-band (150, 400, and 1067 MHz) transmissions, which are used to reconstruct electron density profiles through tomographic techniques [29]. Additionally, magnetometers, particularly fluxgate sensors deployed in networks like LISN, measure variations in the Earth’s magnetic field, which influence the formation and evolution of EPBs and, consequently, the severity of scintillation effects [30].

During the 2000s, SDR technology gained attention as an adaptable and cost-effective alternative for ionospheric monitoring. Unlike conventional receivers, SDR platforms can process GNSS, HF, and VHF signals in real time while allowing for software-based reconfiguration. Moreover, the integration of Cognitive Radio (CR) algorithms enhances the system’s capability to adaptively mitigate scintillation-induced signal degradation, providing a promising approach for future ionospheric research [31].

Considering the increasing dependence on satellite-based systems, the integration of these instruments, ranging from traditional ionosondes to modern SDR architecture, plays a crucial role in advancing ionospheric monitoring. A multi-instrumental approach enhances the accuracy of space weather forecasting and supports the development of mitigation strategies against ionospheric disturbances, ensuring the stability of critical global communication and navigation infrastructures. Table 1 provides a comprehensive overview of the instruments used for ionospheric scintillation monitoring. It includes a brief description of each instrument, its key features, principal monitoring capabilities, and an estimated development cost. The instruments covered comprise GNSS-based receivers, SDR platforms, ionosondes, radio beacons, and auxiliary systems, highlighting their technical characteristics and operational scope.

#### 1.2.1. Cognitive Radio

According to Mitola [37], CR is an example of agent technology in telecommunications; likewise, Mitola apud Albayrak [37] defines an agent as software that exhibits the functional attributes of autonomy, interactivity, reactivity, goal-orientation, mobility, adaptivity, and that is capable of planning, reflection, and cooperation. Besides, Mitolla points out, the term cognitive refers to the mix of declarative and procedural knowledge in a self-aware learning system [37].

The European Telecommunications Standards Institute [38] defines the concept as follows: The CR paradigm has been identified as a promising solution to reconcile the existing conflicts between spectrum demand growth and spectrum underutilization and increase the overall efficiency of spectrum exploitation. Biglieri asserts SDR can adapt its frequencies, modulation/coding schemes, and protocols to increase bandwidth efficiency, while CR has availability of some network side information, and uses it to adapt itself to the surrounding environment, and extends this concept by sensing the radio environment and learning how to adapt its own performance to the user’s needs [39].

In accordance with SDR Forum [40], the definition of CR is presented below:

“(a) Radio in which communication systems are aware of their environment and internal state and can make decisions about their radio operating behavior based on that information and predefined objectives. The environmental information may or may not include location information related to communication systems.

(b) Cognitive Radio (as defined in a.) that utilizes Software-Defined Radio, Adaptive Radio, and other technologies to automatically adjust its behavior or operations to achieve desired objectives.”

Furthermore, Al Hashimi et al. [5] describe that the CR operation cycle includes sensing, analysis, decision, sharing, and adaptation. Each stage has its own challenges with the view of power consumption, speed, throughput, and error probability. Even so, IEEE is working on standardization of the use and operation of the term in projects, i.e., the IEEE 802.22 [41], the Working Group on Wireless Regional Area Networks (“WRANs”). They developed a wide variety of standards to enable spectrum sharing.

As stated in [42], although applications are recognized as in the domain of “CR”, there is much disagreement on exactly what is and what is not a CR, this is due to everyone having their own definition. Concerning showing the attributes that are commonly expected from a CR and from a baseline set of assumptions, the following statement was detailed:

1. Observation: Collect information about the operating environment, capability, and characteristics of the radio.
2. Reconfiguration: Change the operation parameters of the radio.
3. Cognition: Understand the environment and capability of the radio (awareness), make informed decisions on actions (reasoning), and learn the impact of these actions on the performance of the radio, as well as the performance of the network in which the radio is embedded (learning).

Also, Biglieri classifies three paradigms of CR, underlay, overlay, and interweave, according to the operational interaction between users within the spectrum [39]. In the context of CR, two types of users are defined: the Secondary User (SU) and the Primary User (PU) [39]:

- SU: Secondary User: A user that does not hold a license to operate in a specific frequency band, but may opportunistically access it, if it does not cause significant interference to primary users.
- PU: Primary User: A user that is licensed to operate in a specific frequency band and holds full priority over that portion of the spectrum.

The three CR paradigms are defined as follows:

1. Underlay Paradigm: the interference threshold may be defined with respect to a spectral mask, defined as the power spectral density of the interference over the underlay frequency band, or with respect to an average power constraint. As an alternative to determining the interference it causes, a SU may spread its signal over a bandwidth so wide that the interference spectral density it causes to PUs is below a noise threshold.
2. Overlay Paradigm: SUs have knowledge of the PU’s transmitted data sequence and how this sequence is encoded. This knowledge can be exploited by the SU in a variety of ways to improve the performance of its own transmission and that of the PU. A possible strategy is to use the information gathered to cancel the interference due to the primary signals at the secondary receiver. Another strategy consists of apportioning SU power between its own communication and assistance to primary transmissions via relaying.
3. Interweave Paradigm: This is based on the idea of opportunistic communication, one of the original concepts underlying CR. The idea came after the observation that temporary space–time–frequency voids (or “holes”) exist in both the licensed and unlicensed bands. Spectrum holes can be exploited by SUs to operate in orthogonal dimensions of space, time, or frequency relative to PU signals. If this is done, SUs must preliminarily detect PU transmissions in one or more of the space–time–frequency dimensions.

#### 1.2.2. Cognitive Sense

**Figure 4:**
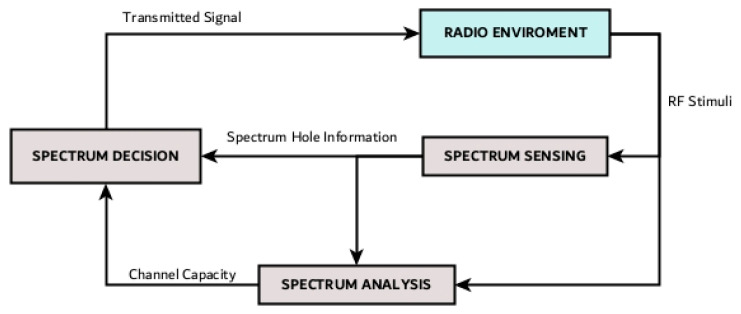
Cognitive cycle. Adapted from [43,44].

CR enables real-time interaction with its environment, allowing it to adapt and determine the most appropriate communication parameters. To achieve this, an adaptive operation is required, as illustrated in Figure 4, in a process known as the cognitive cycle [43]. In this figure, Akyildiz [43] establishes the three basic operational techniques available for all proposed CR systems:

- Spectrum sensing: A cognitive radio monitors the available spectrum bands, gathers information about them, and then detects the spectrum holes. By identifying these unused frequencies, the CR can adapt to its environment. These detection techniques are classified into three categories: transmitter detection, cooperative detection, and interference-based detection.
- Spectrum analysis: The characteristics of the spectrum holes detected through spectrum sensing are estimated. This adaptive process not only requires detecting unused frequencies but also understanding the characteristics of the spectrum bands, which makes it possible to identify the appropriate spectrum band according to user requirements, such as operating frequency, bandwidth, interference level, channel error rate, path-loss, link-layer delay, and holding time.
- Spectrum decision: With the previously identified information, CR determines the data rate, the transmission mode, and the bandwidth of the transmission. Then, the appropriate spectrum band is chosen according to the spectrum characteristics and user requirements.

#### 1.2.3. Historical Evolution of SDR in Ionospheric Scintillation and Cognitive Radio

**Figure 5:**
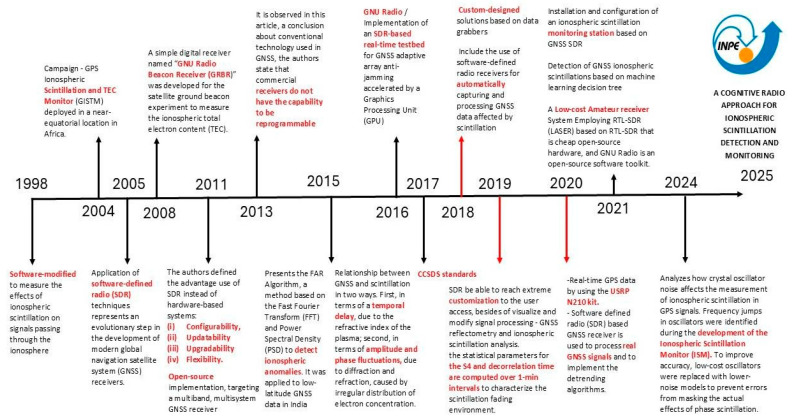
Evolution of technologies for detecting and monitoring ionospheric scintillation. In the figure, the red color represents the highlights of each article and their relationship to the topic studied. Adapted from: [45,46,47,48,49,50,51,52,53,54,55,56,57,58,59,60,61,62,63,64,65,66,67,68].

This section provides a brief historical overview of the technologies for detecting and monitoring ionospheric scintillation, see Figure 5. presents a timeline illustrating the evolution of these technologies in GNSS systems, highlighting key milestones from 1998 to 2025.

- 1998–2004: Initial research begins with software modifications to measure the effects of scintillation on GNSS signals.
- 2005–2011: The development of open-source digital receivers, such as the GNU Radio Beacon Receiver (GRBR), enables advancements in measuring the TEC.
- 2013–2015: The limitations of commercial GNSS receivers are identified, particularly their lack of reprogrammability. Meanwhile, methods like the FAR Algorithm are introduced for ionospheric spectral analysis.
- 2016–2019: The relationship between GNSS and ionospheric scintillation is established, leading to the adoption of Consultative Committee for Space Data Systems (CCSDS) standards to enhance customization and visualization of GNSS signals. Additionally, Software-Defined Radio (SDR)-based solutions are implemented for real-time monitoring.
- 2020–2021: Ionospheric scintillation monitoring stations are developed using low-cost receivers such as the Low-cost Amateur Receiver System (LASER), incorporating machine learning algorithms for event detection.
- 2024–2025: Improvements in GNSS receiver crystal oscillators are analyzed to reduce errors and increase accuracy, culminating in the development of the Ionospheric Scintillation Monitor (ISM) as an innovative cognitive radio–based solution.

### 1.3. EPB Occurrence Detection in Colombia

**Figure 6:**
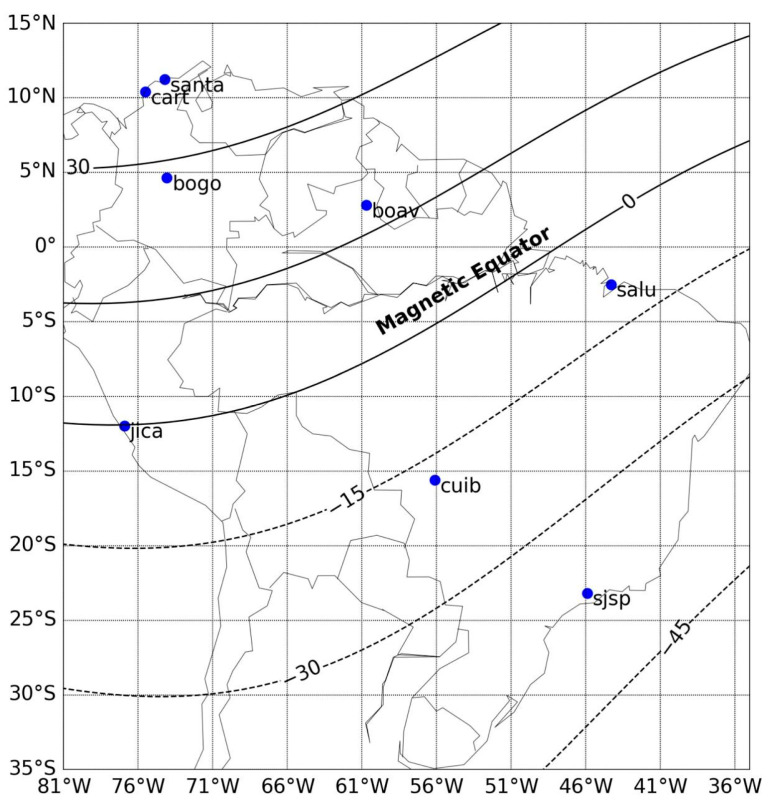
Chosen locations in Colombia.

Due to its geographic location near the magnetic equator, see Figure 6, Colombia holds a unique position for studying both low- and Middle-latitude scintillations, particularly the transition region between these two zones. In this region, scintillation exhibits distinct characteristics, such as weak but very often occurring frequency. This phenomenon is associated with Medium Scale Traveling Ionospheric Disturbances (MSTIDs) through Perkins instability, the neutral wind, and electrical fields through sporadic E (Es) layer instability [15,16]. And with this privileged location, Colombia has the potential to study Middle-latitude scintillation, a region that has been scarcely studied. Despite this, the country lacks a robust monitoring network for real-time observation of these phenomena.

Countries such as Brazil have deployed nationwide monitoring networks to enable continuous observation of ionospheric scintillation phenomena. Since February 2011, Brazil has operated such a system under the CIGALA (Concept for Ionospheric Scintillation Mitigation for Professional GNSS in Latin America) and CALIBRA (Countering GNSS High Accuracy Applications Limitations due to Ionospheric Disturbances in BRAzil) projects, collectively known as the CIGALA/CALIBRA network [69]. This infrastructure provides continuous measurements of ionospheric TEC and scintillation parameters such as the S_4_ index through advanced GPS/GNSS receiver networks and VHF monitoring stations.

**Figure 7:**
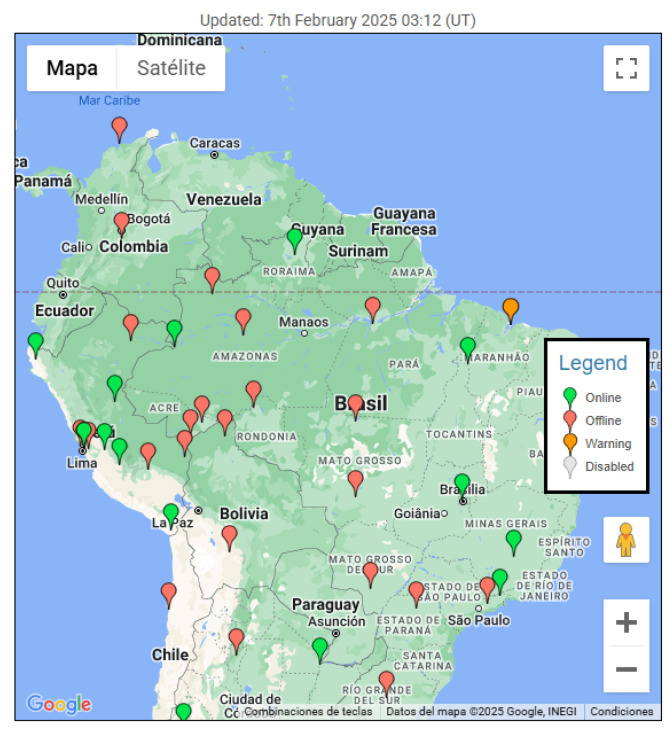
Low-latitude ionospheric sensor network (2025). The gray dashed line represents the geographic equator [71].

In contrast, as illustrated in Figure 7, Colombia faces a significant gap in its observational capabilities, limiting its contribution to international space weather research efforts [70]. Moreover, this lack of infrastructure increases the vulnerability of the country’s critical systems to operational disruptions caused by intense scintillation events, due to insufficient detection and mitigation mechanisms.

To address this issue, this study proposes the development of a Low-Cost Scintillation Laboratory (LCSL) based on SDR and CR technology. This system will enable real-time detection, monitoring, and warning of ionospheric scintillation in Colombia. SDR technology provides a highly configurable and flexible platform for continuous ionospheric observation, while the incorporation of artificial intelligence (AI) algorithms based on deep learning allows the use of data from the Jicamarca Radio Observatory as validation in a conjugate pairs example. The application of ML models in EPBs detection will enhance the system’s predictive capabilities and minimize false alarms, representing a substantial improvement over the conventional fixed-threshold approach [72].

The LCSL will be implemented across three monitoring stations located in Bogotá, Cartagena, and Santa Marta, see Figure 6 in Section 1.3, strategically selected for their proximity to the northern crest of the EIA. The proposed system will utilize GNSS signal data in the L1 band (1575.42 MHz) and VHF (145.95 MHz) to characterize ionospheric dynamics under both geomagnetically quiet and disturbed conditions. Additionally, a Systems Engineering framework will be implemented to define the laboratory’s functional and operational requirements, ensuring scalability and efficiency in data acquisition and processing.

This study not only aims to bridge the technological gap between commercial monitoring systems and the need for accessible tools for ionospheric research in Colombia but also contributes to advancements in space weather knowledge. By providing an innovative and cost-effective solution for scintillation monitoring, this research will strengthen the country’s capacity to address challenges associated with GNSS signal degradation and its impact on critical navigation and communication applications. Finally, the findings of this study may serve as a foundation for future policy recommendations on space weather monitoring in the region, promoting greater integration of Colombia into international research efforts, and on strategies for ionospheric effects on navigation and telecommunications systems.

The remainder of this paper is organized as follows: Section 2 details the development and architectural design of the LCSL system, describing the methodological stages of the investigation. Section 3 describes the implementation results in Colombia, examines operational constraints, and presents comparative analyses including cognitive radio algorithm evaluation, DIMSUMnet versus Adapt4 XG1 performance assessment, experimental data validation, and machine learning integration. Section 4 concludes the study by summarizing the principal contributions and identifying future research directions.

## 2. Materials and Methods

The methodology employed in this research follows an applied research approach within the Design Science Research (DSR) paradigm, which focuses on creating innovative artifacts to solve real-world problems [73]. This approach was chosen to develop a low-cost and scalable ionospheric scintillation monitoring system by integrating SDR, CR, and ML techniques. To ensure a structured and systematic development, the study adopts the Systems Engineering Process (SEP) as its guiding framework [74]. SEP is utilized to define system requirements, analyze functional architectures, and optimize adaptive signal processing techniques for real-time space weather analysis. This methodological combination allows for the design, implementation, and validation of an intelligent monitoring system that can enhance the accuracy and reliability of ionospheric scintillation detection while remaining cost-effective and adaptable for deployment in resource-limited regions.

### 2.1. Phases of LCSL Development

**Figure 8:**
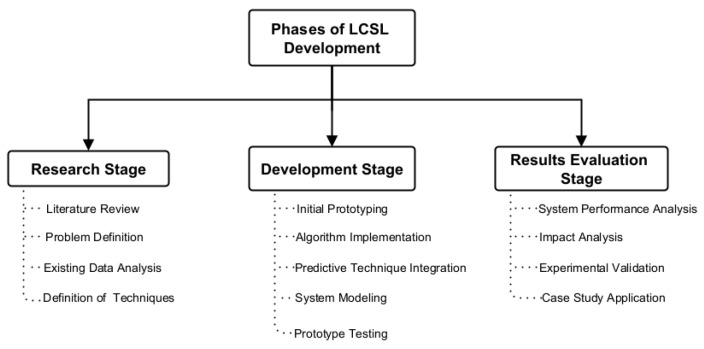
Phases of LCSL development.

The development of the LCSL followed a structured methodological approach, ensuring systematic progression from research to implementation and evaluation. This process was divided into three approaches, which, along with their sub-phases, are illustrated in Figure 8.

#### 2.1.1. Research Stage

In this stage, a comprehensive analysis of relevant literature was conducted, alongside the selection of appropriate methodologies to support both problem definition and solution development. The study follows an applied research framework designed to generate practical knowledge. To address the problem effectively, the DSR paradigm was adopted, emphasizing the advancement of human knowledge through the creation of innovative artifacts. The phase was organized as follows:

- Literature review: Academic publications related to operational simulators, GNSS communication systems, and their vulnerabilities to ionospheric scintillation were explored.
- Problem definition: The methodology ‘Processo de Referência para o Desenvolvimento da Arquitetura de uma Estação Terrena para Pico e Nanosatélites’ was applied to structure the system’s architecture, identify critical subsystems, and define the requirements and constraints for the system’s development.
- Existing data analysis: The study adopted the Cross-Industry Standard Process for Data Mining (CRISP-DM) model, complemented with machine learning techniques, including YOLO-trained neural networks for real-time analysis of ionospheric disturbances. These methods were validated using historical data from the IGP and international GNSS networks.
- Definition of predictive and mitigation techniques: A comprehensive assessment was conducted to identify and integrate specific methods for predicting and mitigating the effects of scintillation on GNSS links. This involved evaluating statistical models, deep learning–based classification techniques, and adaptive filtering methods to improve signal resilience under adverse ionospheric conditions.

#### 2.1.2. Development Stage

This stage focused on designing, implementing, and testing a cognitive radio system to mitigate scintillation effects. The following activities were carried out:

- Initial prototyping: An experimental model in LabVIEW 2023 was designed for detecting scintillation conditions.
- Implementation of cognitive radio algorithms: Algorithms were developed to enhance the detection and monitoring of scintillation events in multiband links (VHF and L-band).
- Integration of predictive techniques: ML models and statistical methods were implemented to predict scintillation occurrence, integrating YOLO-based neural networks, which provided real-time analysis for improved accuracy.
- System modeling: A GNSS communication model was developed in MATLAB (2025 for Academic use), including interference scenarios caused by EPBs, to validate the robustness of the algorithms.
- Prototype testing: Rigorous tests were conducted under controlled conditions to evaluate its performance before applying it to case studies.

**Figure 9:**
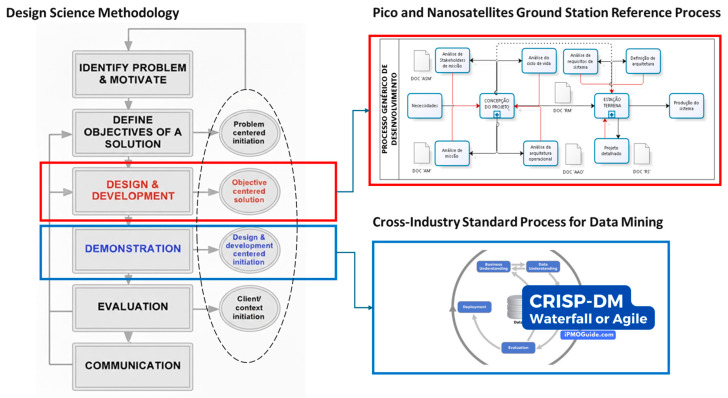
Methodology research design, source: Adapted from [74,75].

In Figure 9, the research and development stages can be seen, along with how they complement each other. This adapted methodology allows, based on existing data and emerging technologies, to contribute to the advancement of current technologies by reducing production costs and enabling the development of low-cost solutions, thereby increasing opportunities for scintillation investigation.

#### 2.1.3. Results Evaluation Stage

This phase aimed to validate the system’s effectiveness in real-world scenarios by systematically evaluating its performance and impact.

To perform this stage, the following steps were realized:

- System performance analysis: The accuracy, sensitivity, and specificity of the developed algorithms were measured and optimized with the goal of enhancing their performance for ionospheric scintillation detection.
- Impact analysis: The effects of EPBs on GNSS links were modeled, and the proposed mitigation strategies were evaluated.
- Experimental validation: The techniques and prototypes were applied in real-world scenarios to demonstrate the feasibility of cognitive radio systems in space weather studies.
- Case study application: Validation tasks were conducted in Bogotá, Cartagena, and Santa Marta, demonstrating the system’s capability to operate effectively under diverse geographical and ionospheric conditions.

### 2.2. Tools and Technologies Used

The development of this study required the use of various technological tools:

- Software-Defined Radio (SDR): USRP B210, a low-cost SDR hardware, was used for GNSS signal acquisition and processing.
- MATLAB: Used for digital signal processing, data modeling, and simulation of ionospheric interference scenarios.
- Python 3.10 and TensorFlow 2.18.0: ML models were implemented using these frameworks, including YOLO neural networks for real-time scintillation detection.
- LabVIEW: This tool facilitated experimental prototyping and validation of CR algorithms, allowing for streamlined system testing.
- Google Colab: The cloud-based computing environment was leveraged for model training and optimization, taking advantage of high-performance GPU resources.

By integrating these methodologies and tools, this study aims to improve the detection, analysis, and mitigation of ionospheric scintillation, contributing to the reliability of GNSS navigation and space weather monitoring in Colombia.

### 2.3. LCSL System Hardware Configuration and Software Integration Architecture

**Figure 10:**
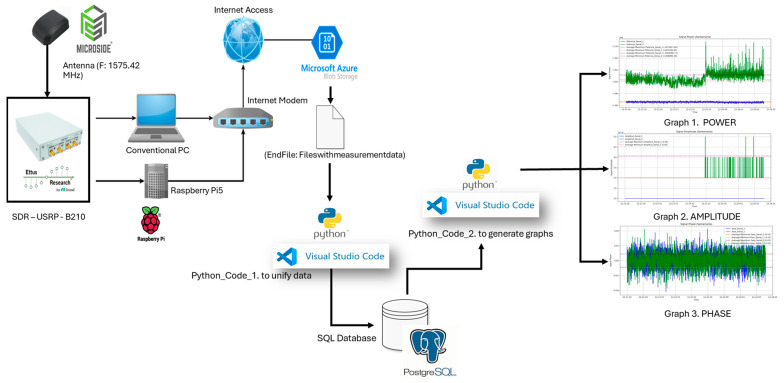
LCSL system hardware and software integration architecture.

As illustrated in Figure 10, the LCSL system is structured as an end-to-end SDR-based acquisition, processing, and data management platform integrating radio frequency capture, edge computing, centralized processing, and database storage within a unified operational framework.

At the hardware level, the signal chain begins with the GNSS multiband antenna and RF front-end, which capture L-band signals and feed them into a USRP B210 software-defined radio. The SDR performs digitization of complex IQ samples and transfers high-throughput data streams to the host system through a USB 3.0 interface. Edge-level devices, including a Raspberry Pi 4 Model B with 4 GB of RAM (It was created by Eben Upton and developed by the Raspberry Pi Foundation in Cambridge, United Kingdom. It is a British-designed device manufactured in the United Kingdom) and a conventional workstation, manage preliminary acquisition control and local buffering before forwarding structured datasets through the network interface toward centralized processing modules.

To maintain operational stability under non-stationary ionospheric conditions, the acquisition subsystem incorporates adaptive hardware control. Receiver gain, sampling rate, acquisition bandwidth, and center frequency are dynamically adjusted according to real-time estimates of signal-to-noise ratio (SNR), received power, classifier confidence, and computational load. This closed-loop mechanism ensures signal integrity, prevents buffer saturation, and preserves computational stability across varying disturbance regimes.

The software architecture follows a modular hybrid MATLAB–Python integration model, as depicted in Figure 10. MATLAB manages SDR configuration, continuous IQ acquisition, digital pre-processing, and feature extraction, including spectral metrics, power statistics, and scintillation indicators. Extracted signal segments are encapsulated into structured data containers and transferred to the machine learning environment implemented in Python (TensorFlow), where classification or regression inference is executed. Communication between modules is implemented through structured exchange formats and synchronized metadata, ensuring deterministic interoperability.

Processed outputs, together with raw data and derived features, are stored in a PostgreSQL database to guarantee traceability, dataset consistency, and future retraining capability. Data transfer between modules operates under an asynchronous producer-consumer scheme with timestamp synchronization, integrity validation, and support for both streaming and batch processing modes. Latency control is achieved through circular acquisition buffers and sliding processing windows, enabling near real-time operation.

Feedback from the inference layer to the acquisition subsystem enables dynamic system adaptation. When reduced classification confidence or anomalous signal behavior is detected, the system can increase temporal resolution, adjust receiver gain under low-SNR conditions, reduce sampling rate during high computational load, or trigger extended recording intervals for persistent disturbances.

## 3. Results and Discussion

Firstly, this work highlights the limited infrastructure currently available in Colombia for monitoring ionospheric scintillation, a condition primarily attributed to the high costs associated with the implementation, operation, and maintenance of specialized ground-based stations. At present, only four sensors belonging to the Low-Latitude Ionospheric Sensor Network (LISN) are installed within Colombian territory. This network comprises observatories specifically designed to perform nowcasting and forecasting of scintillation conditions, providing critical data for the study and understanding of the dynamics of ionospheric irregularities in the equatorial region.

The four monitoring centers are in Bogotá, Leticia, Popayán, and Santa Marta. However, currently, only the station in Leticia remains operational, which limits the spatial and temporal coverage of the data available for characterizing ionospheric behavior in Colombia. This gap presents a barrier to the development of national capabilities in space weather monitoring, impacting both scientific research and the advancement of more robust satellite navigation systems against ionospheric disturbances.

Despite the limited number of monitoring centers in Colombia, the National University of Colombia operates a station called BOGT00COL, which is part of the International GNSS Service (IGS) network. This station enables both ionospheric and geodetic monitoring of the region, allowing for the observation of ionospheric disturbances, atmospheric effects, and relevant geophysical processes.

Using the data obtained from this station, the ROTI (Rate of TEC Index) is evaluated. This parameter measures the rate of change of the Total Electron Content (TEC) over time for each of the stations analyzed. ROTI enables the detection of ionospheric irregularities that may affect the performance of GNSS systems, as such irregularities can distort the propagation of satellite signals, compromising the accuracy and reliability of positioning services.

### 3.1. Ionospheric Behavior in the Monitoring Stations

#### 3.1.1. Bogotá (13 December 2024)

**Figure 11:**
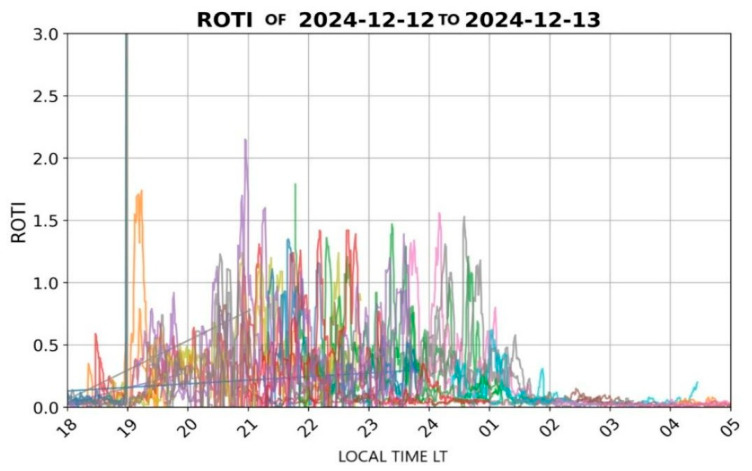
ROTI derived from GNSS observations at IGS station BOGT00COL on Day of Year (DOY) 348 (12–13 December 2024). The x-axis represents LT (UTC-5), and the y-axis shows ROTI values. Each curve corresponds to an individual satellite track. Source: Adapted from [76].

In Bogotá, on the selected date (13 December 2024), significant fluctuations were recorded in the GNSS L1 band during sunset, as illustrated in Figure 11. During this period, pronounced peaks in ROTI values were observed between 19:00 and 01:00 local time (LT). These elevated ROTI values suggest the presence of ionospheric irregularities, such as plasma bubbles, which compromise the stability and accuracy of satellite-based positioning systems.

**Figure 12:**
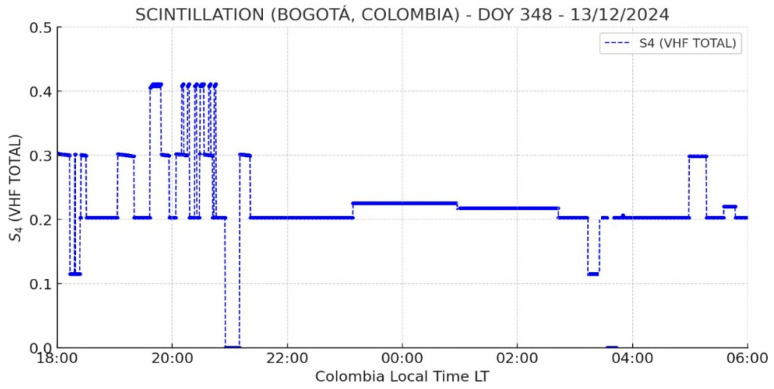
Temporal evolution of the S4 scintillation index measured at the BOG VHF LCSL station in Bogotá, Colombia, on 13 December 2024 (DOY 348). The horizontal axis represents local time (LT, UTC-5). Solid lines correspond to station-recorded measurements, while shaded areas are included to enhance visualization and facilitate interpretation of variability patterns. Data source: authors’ observations.

Figure 12 shows the variation of the S_4_ index between 18:00 and 06:00 LT, with noticeable fluctuations before 22:00 LT and more stable values afterward. This pattern suggests the presence of ionospheric disturbances over Bogotá during the early hours of the observed period, with S_4_ values ranging from 0.019 to 0.4. However, the gap in the interpolation between 01:00 and 06:00 LT indicates a possible signal loss or anomalous reception, likely caused by transient interference, equipment limitations, or environmental conditions affecting VHF signal propagation.

**Figure 13:**
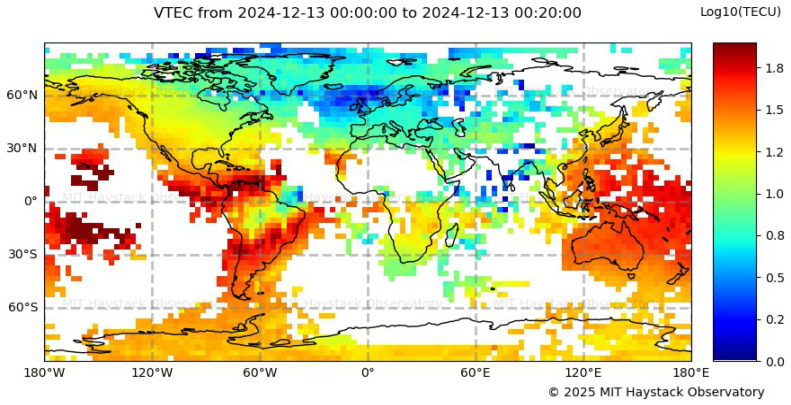
Global distribution of VTEC on 13 December 2024 between 00:00 and 00:20 UTC. The map presents GNSS-derived VTEC values in Total Electron Content Units (TECU) on a logarithmic scale. Source: [77].

The variation of the S_4_ index observed over Bogotá during the specified period indicates a certain degree of ionospheric disturbance. However, since this fluctuation was detected during a technological test, it is possible that it resulted from system-induced distortions rather than genuine ionospheric scintillations. For this reason, the Global Vertical Total Electron Content (VTEC) Map, shown in Figure 13, is analyzed, revealing a high variation in TEC levels. The combined presence of ROTI variations and elevated TEC values suggests a high probability of moderate-to-strong scintillation events over Bogotá. This confirms that the region underwent a period of ionospheric disturbances, as further supported by the low S_4_ index values recorded during that time.

**Figure 14:**
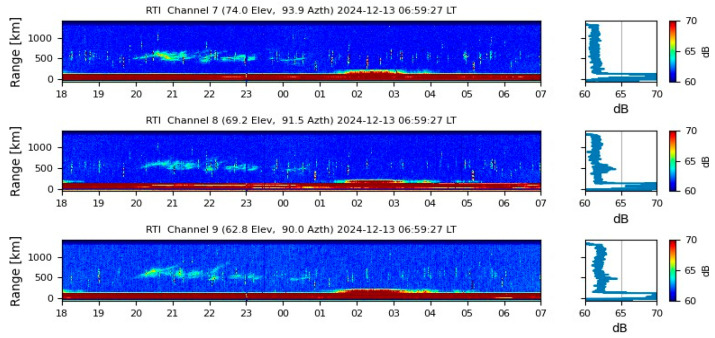
IGP, RTI plots from observations on 13 December 2024 (18:00–07:00 LT, UTC-5). Channels 7–9 correspond to different elevation and azimuth angles, showing temporal (x-axis, LT, UTC-5) and range-dependent variations in received signal intensity (dB), indicative of possible ionospheric irregularities. Data source: Adapted from [78].

On the other hand, Figure 14 presents a Range-to-Intensity (RTI) map extracted from Incoherent Scatter Radar measurements at the Jicamarca Observatory. Signatures of plasma bubbles are evident between 20:00 and 23:00 LT. Meanwhile, the S_4_ GNSS index recorded in Bogotá fluctuated between 0.019 and 0.4, indicating moderate perturbations throughout the observation period. The significant post-sunset variations observed in Bogotá closely align with the moderate irregularities detected in Jicamarca, suggesting that the ionosphere underwent a period of moderate plasma turbulence.

**Figure 15:**
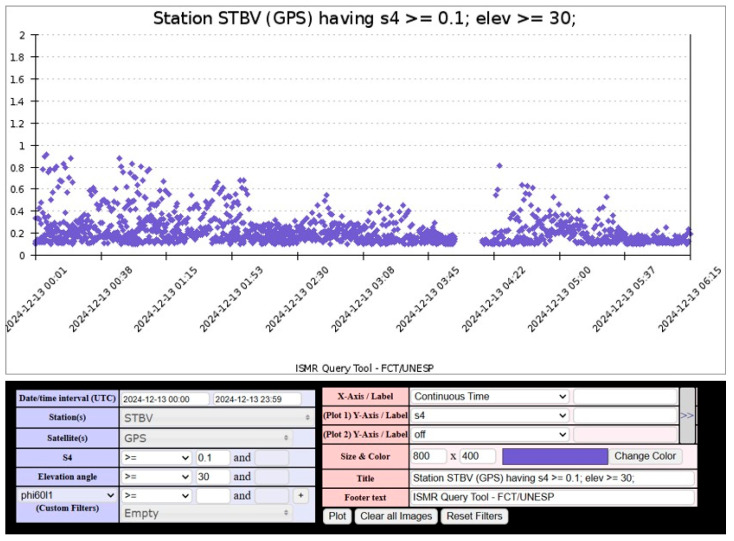
GPS S_4_ scintillation index measured at station STBV on 13 December 2024. The scatter plot shows S_4_ values from GPS observations for satellite links with elevation angles ≥ 30° and S_4_ ≥ 0.1, plotted as a function of continuous time. Data was obtained using the CIGALA/CALIBRA monitoring system. Source: authors.

The comparison between CIGALA/CALIBRA data and the observations from the Bogotá station on 13 December 2024 reveals a correlation between ionospheric scintillation patterns recorded in Bogotá and Boa Vista (Brazil). In both cases, the S_4_ index exhibited marked variability at the beginning of the period, indicating significant fluctuations in GNSS signals, which subsequently stabilized, as shown in Figure 15. This similarity suggests that the ionosphere over Bogotá underwent substantial perturbations, thereby confirming the presence of localized ionospheric irregularities.

#### 3.1.2. Cartagena (17–18 December 2024)

**Figure 16:**
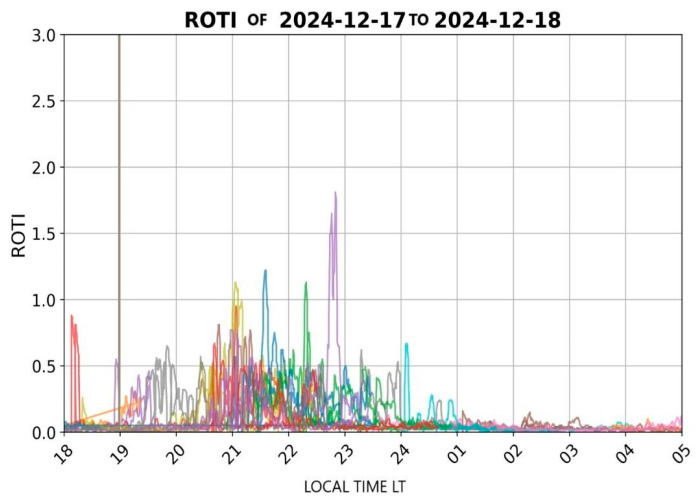
ROTI derived from GNSS observations at IGS station BOGT00COL on DOY 352 (17–18 December 2024). The x-axis represents LT (UTC-5), and the y-axis shows ROTI values. Each curve corresponds to an individual satellite track. Source: adapted from [76].

In Cartagena, on the selected dates (17–18 December 2024), significant fluctuations were recorded in the GNSS L1 band during sunset, as illustrated in Figure 16. During this period, pronounced peaks in ROTI values were observed between 19:00 and 23:00 LT. These elevated ROTI values suggest the presence of intense ionospheric irregularities, such as equatorial plasma bubbles, which can affect the stability and accuracy of satellite-based positioning systems. Although there is no dedicated GNSS receiver in Cartagena, data from the IGS GPS station in Bogotá was utilized to assess the ionospheric behavior over the region, leveraging the geographical proximity between both locations. This preliminary analysis highlights the regional coherence of low-latitude ionospheric disturbances and supports the development of improved ionospheric detection models in Colombia.

**Figure 17:**
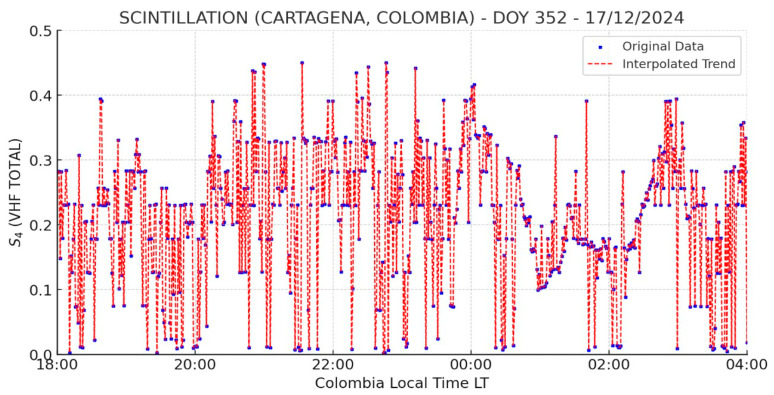
Temporal variation of the S_4_ scintillation index measured at the CTG VHF LCSL station in Cartagena, Colombia, on 17 December 2024 (DOY 352). The figure shows S_4_ values derived from VHF observations as a function of LT (UTC-5). Data source: authors’ observations.

Figure 17 presents the results obtained for the S_4_ index at the monitoring station located in Cartagena. The figure shows the monitoring period between 18:00 and 04:00 LT, during which the most significant fluctuations in the S_4_ index occur between 20:00 and 00:00 LT, with values ranging from 0.1 to 0.45. This behavior indicates an increase in scintillation activity during this time frame. After midnight, between 00:00 and 03:00 LT, the data reflect a relatively stable pattern, suggesting low scintillation conditions. However, it is important to consider the possibility of instrumental noise, as a final peak in the S_4_ index is observed after 04:00 LT.

**Figure 18:**
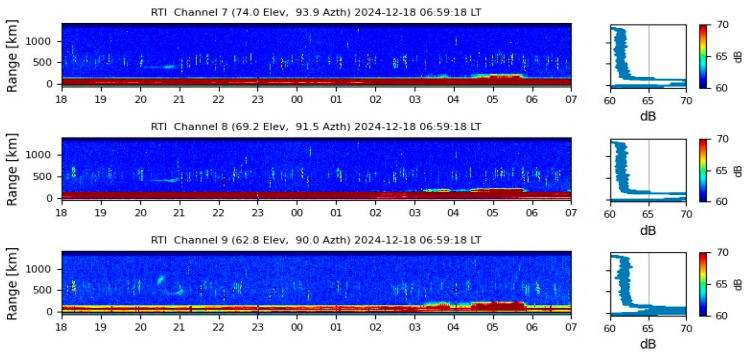
IGP, RTI plots from observations on 18 December 2024 (18:00–07:00 LT, UTC-5). Channels 7–9 correspond to different elevation and azimuth angles, showing temporal (x-axis, LT, UTC-5) and range-dependent variations in received signal intensity (dB), indicative of possible ionospheric irregularities. Data source: Adapted from [78].

The fact that the highest S_4_ index values in Figure 18 were recorded between 20:00 and 00:00 LT aligns with previous observations regarding the onset of scintillation phenomena following sunset. However, during the same period, low levels of scintillation were reported at the Jicamarca Observatory, as shown in Figure 18. This discrepancy suggests that the Cartagena station may be capturing excessive noise in the VHF band, highlighting the importance of validation data and effective noise filtering strategies in ionospheric monitoring. Despite the system’s susceptibility to noise, it is worth noting that Jicamarca did detect mild scintillation events between 20:00 and 22:00 LT that were recorded with greater intensity by the Cartagena station, likely amplified by the noise component.

**Figure 19:**
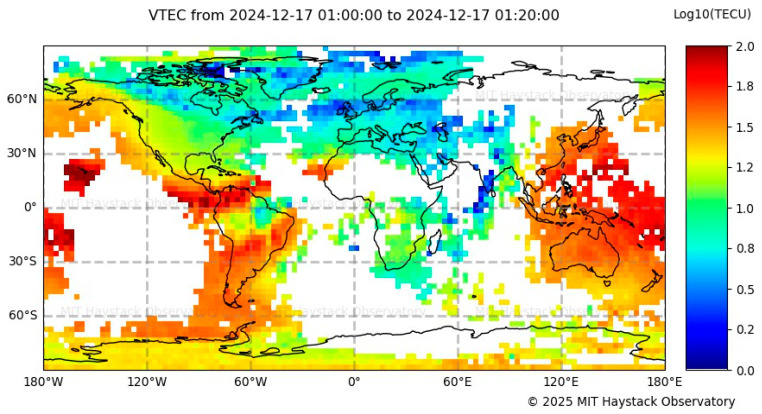
Global distribution of VTEC on 17 December 2024, between 00:00 and 00:20 UTC. The map presents GNSS-derived VTEC values in Total Electron Content Units (TECU) on a logarithmic scale. Source: [77].

The variation of the S_4_ index observed over Cartagena during the specified period indicates the presence of ionospheric disturbances, with the highest activity recorded between 20:00 and 00:00 LT. To further investigate this behavior, the Global VTEC Map, shown in Figure 19, is analyzed, revealing elevated TEC values over the equatorial region of Colombia at 01:00 UTC (20:00 LT, UTC-5; in Cartagena), along with medium TEC values over Jicamarca. The pronounced TECU levels centered over Colombia suggest a well-developed EIA, with one of its crests located above the region. The combined evidence of S_4_ fluctuations and high TEC values points to a high probability of strong scintillation events over Cartagena, consistent with the known tendency for ionospheric scintillation to intensify at the crests of the EIA.

**Figure 20:**
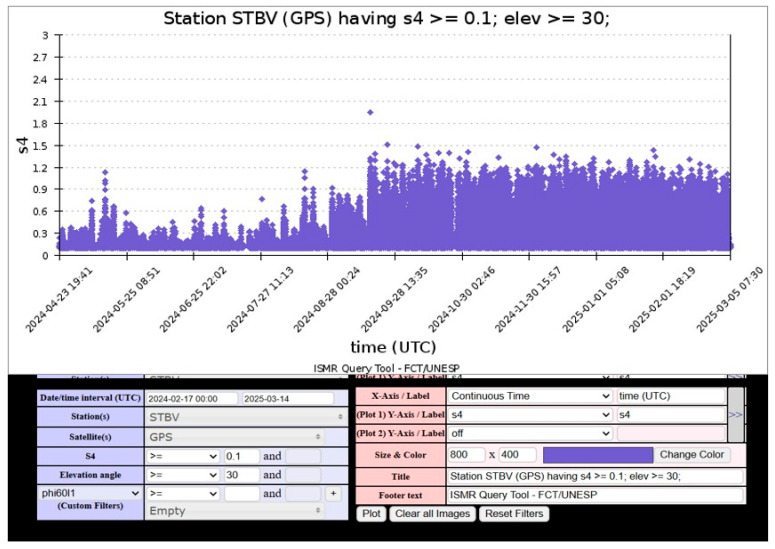
GPS S_4_ scintillation index measured at station STBV on 17 December 2024. The scatter plot shows S_4_ values from GPS observations for satellite links with elevation angles ≥ 30° and S_4_ ≥ 0.1, plotted as a function of continuous time. Data was obtained using the CIGALA/CALIBRA monitoring system. Source: authors.

The comparison between CIGALA/CALIBRA data and the observation from Cartagena reveals a correlation between ionospheric scintillation patterns recorded in Cartagena and Boa Vista (Brazil). In both cases, the S_4_ index exhibited sustained variability during the nighttime period, indicating prolonged ionospheric irregularities, as shown in Figure 20. In Boa Vista, S_4_ values reflect continuous scintillation from 00:24 to 07:30 (UTC-4), while in Cartagena, scintillation activity begins around 23:00 LT, consistent with post-sunset equatorial plasma instabilities and the eastward drift of irregularities. The observed increase in S_4_ values between 00:00 and 01:00 (UTC-4), with peaks exceeding 1.5, suggests intense scintillation conditions that can significantly degrade GNSS performance and disrupt VHF signal propagation.

The scintillation analysis for 17 December 2024 indicates moderate ionospheric activity over Cartagena, with S_4_ index values ranging from 0.14 to 0.45 during the period from 20:00 to 00:00 LT. Initially, values remained low between 00:00 and 03:00 LT, followed by a peak exceeding 0.40, suggesting an increase in ionospheric irregularities. In contrast, the CIGALA/CALIBRA network recorded sustained scintillation from 00:00 to 07:30 LT, with S_4_ values exceeding 1.5, indicative of intense ionospheric disturbances. Meanwhile, the IGP Jicamarca Observatory reported only minimal variations between 20:00 and 22:00 LT, suggesting relatively quiet ionospheric conditions in that region. Additionally, the MIT/Haystack Observatory reported very high TEC values over Colombia between 01:00 and 07:30 UTC, further supporting the likelihood of ionospheric perturbations during this period. The correlation among these datasets validates the scintillation records from Cartagena, confirming that the observed fluctuations were caused by ionospheric disturbances.

#### 3.1.3. Santa Marta (24 December 2024)

**Figure 21:**
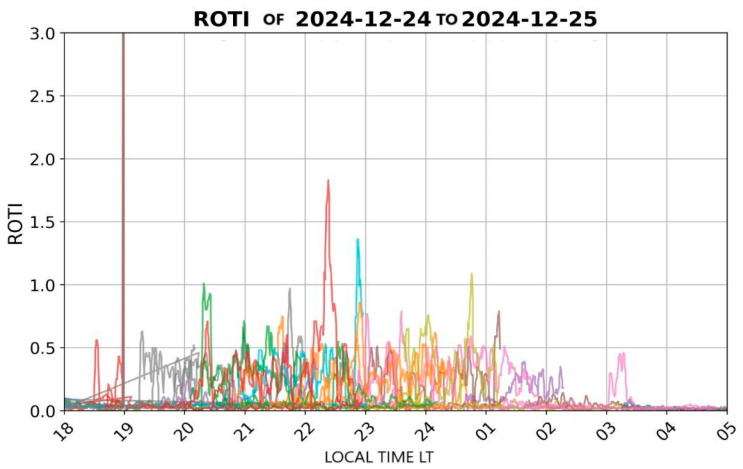
ROTI derived from GNSS observations at IGS station BOGT00COL on DOY 359 (24–25 December 2024). The x-axis represents LT (UTC-5), and the y-axis shows ROTI values. Each curve corresponds to an individual satellite track. Source: adapted from [76].

In Santa Marta, on the selected date (24 December 2024), significant fluctuations were recorded in the GNSS L1 band after sunset, as illustrated in Figure 21. During this period, pronounced peaks in ROTI values were observed between 19:00 and 23:30 LT. These elevated ROTI values indicate strong fluctuations in electron density, suggesting the presence of ionospheric scintillation phenomena that can affect the performance of VHF signals. Due to the absence of a dedicated GNSS receiver in Santa Marta, data from the IGS GPS station in Bogotá were utilized, given the geographical proximity between both locations. This approach allows for a preliminary analysis of ionospheric behavior over the Santa Marta region and supports regional assessments, considering that ionospheric disturbances in low-latitude areas often exhibit spatial coherence.

**Figure 22:**
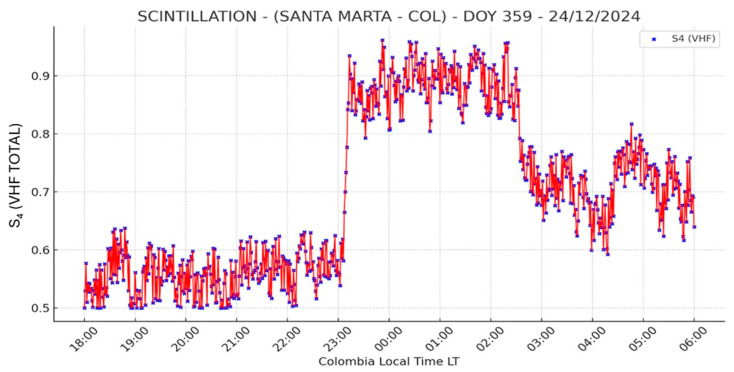
Temporal variation of the S_4_ scintillation index measured at the STM VHF LCSL station in Santa Marta, Colombia, on 17 December 2024 (DOY 359). The figure shows S_4_ values derived from VHF observations as a function of LT (UTC -5), the red line represents the temporal evolution of S_4_. Data source: authors’ observations.

As shown in Figure 22, this station recorded the highest scintillation activity, primarily due to its proximity to the northern crest of the IA. The elevated S_4_ index values clearly indicate extreme scintillation conditions, which can severely affect GNSS signal quality. This behavior was expected, given the station’s location near the EIA, where higher plasma density enhances scintillation effects. Several points reached S_4_ values close to 0.9, indicating strong scintillation conditions, with peak activity occurring around 23:00 and 03:00 LT, reaching values near 1.0. Following these peaks, a decrease in the S_4_ index was observed between 03:00 and 06:00 LT, with values ranging between 0.6 and 0.8. This further supports the presence of sustained scintillation activity in the region.

The high S_4_ index values recorded during the selected interval and date suggest the occurrence of intense scintillation events. However, it is essential to implement a validation mechanism to ensure that the observed fluctuations are indeed attributable to ionospheric scintillation and not to monitoring system interference or anomalies.

**Figure 23:**
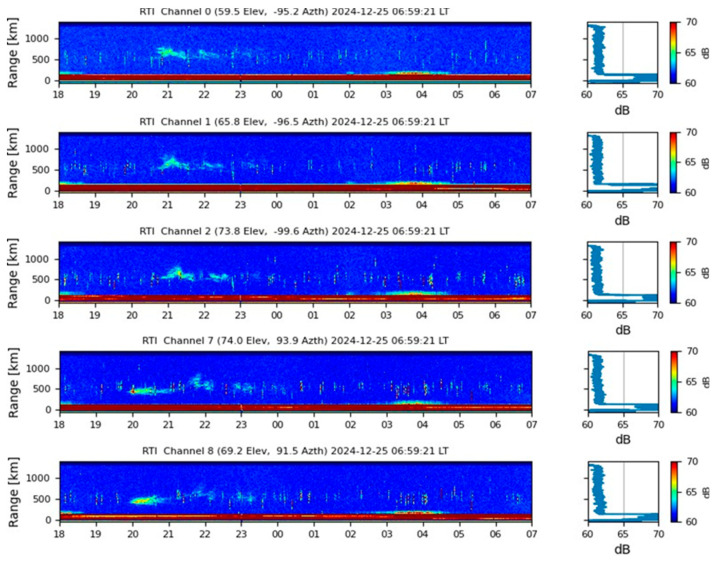
IGP, RTI plots from observations on 25 December 2024 (18:00–07:00 LT, UTC-5). Channels 0–2 and 7–8 correspond to different elevation and azimuth angles, showing temporal (x-axis, LT, UTC-5) and range-dependent variations in received signal intensity (dB), indicative of possible ionospheric irregularities. Data source: Adapted from [78].

In comparison, scintillation data from the Jicamarca Observatory, shown in Figure 23, also reported ionospheric irregularities between 20:00 and 00:00 LT, reinforcing the spatial coherence of equatorial ionospheric activity. This alignment suggests that favorable conditions for plasma instability development were present across the region. The ability to observe strong scintillation in both Santa Marta and Jicamarca highlights the importance of multi-site and multi-frequency monitoring to ensure accurate characterization of ionospheric disturbances.

**Figure 24:**
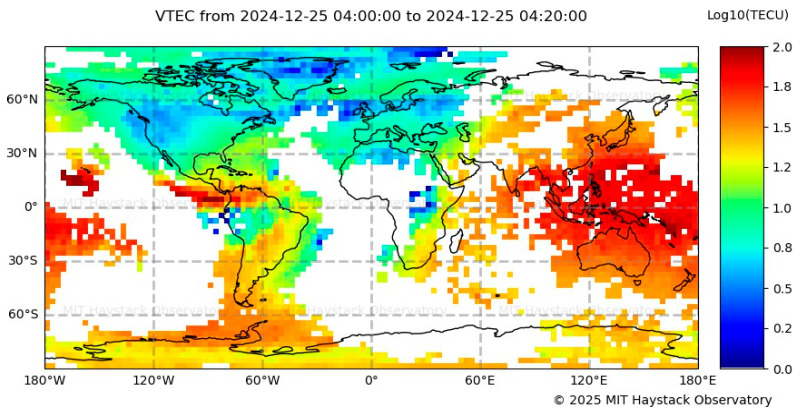
Global distribution of VTEC on 25 December 2024, between 00:00 and 00:20 UTC. The map presents GNSS-derived VTEC values in Total Electron Content Units (TECUs) on a logarithmic scale. Source: [77].

Consistent with previous observations, the Global TEC Map shown in Figure 24 reveals elevated TEC levels over Colombia between 04:00 and 04:20 UTC. These significantly high values, particularly over the Santa Marta region (±20° magnetic latitude), confirm a high plasma density, indicating the presence of ionospheric irregularities that led to severe scintillation conditions. This comparison confirms that the elevated plasma content in the region contributed to the intense scintillation observed in Santa Marta. However, due to the magnitude of the recorded S_4_ values, further analysis, including multi-frequency GNSS observations and noise filtering techniques, is required to robustly validate the dataset.

Scintillation analysis on 24 December 2024 shows extreme ionospheric activity in Santa Marta, with S_4_ values peaking near 1.0 between 23:00 and 03:00 LT, indicating severe GNSS signal disruptions. In contrast, IGP Jicamarca observed moderate activity (20:00–23:00 LT). MIT/Haystack Observatory reported very high TEC values (04:00–04:20 UTC), reinforcing strong plasma irregularities over the region.

### 3.2. Systems Engineering Process Results

The SEP approach has demonstrated its effectiveness as a structured and repeatable methodology, ensuring high adaptability, accuracy, and seamless integrations with international monitoring systems. Additionally, it provides scalability, modularity, and risk mitigation in GNSS signal analysis, particularly in environments affected by ionospheric disturbances.

When combined with the SDR approach, which offers a highly configurable and flexible platform for continuous ionospheric sounding during both geomagnetically calm periods and space weather events, the system becomes even more robust. Furthermore, with advanced power management and AI-based processing, this approach enhances the detection and understanding of scintillation effects on communication and navigation systems.

**Figure 25:**
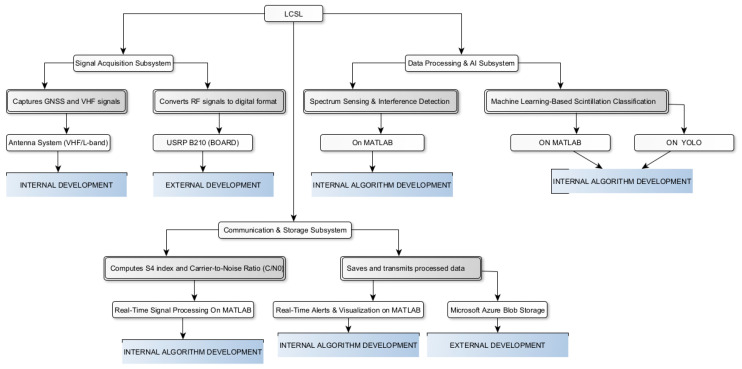
Low-cost scintillation laboratory (LCSL), source: Authors.

LCSL employs a cognitive radio approach, allowing it to dynamically adapt to signal conditions through real-time spectrum sensing, interference mitigation, and adaptive frequency selection. Its architecture integrates SDR with AI models for spectral analysis and signal classification, ensuring resilience against environmental and interference factors. This structured methodology enables real-time detection, secure data transmission, and compliance with international GNSS monitoring standards, establishing LCSL as an essential tool for ionospheric research. Figure 25 illustrates the LCSL structure.

Several factors influence the development and operation of LCSL. Among the technical constraints, hardware limitations, software scalability, and computational power requirements stand out. Additionally, environmental factors, such as electromagnetic interference, weather conditions, and geographical variations, impact GNSS signal quality, necessitating adaptive system configurations. Logistical challenges, including power supply availability and network connectivity, require alternative solutions such as solar-powered infrastructure and satellite-based communication. Furthermore, the laboratory must comply with national and international regulations to ensure alignment with global space weather monitoring standards, fostering worldwide research collaboration.

### 3.3. Development and Implementation of a Scintillation Visualization System for Bogotá: A Scalable Approach for Nationwide Monitoring

**Figure 26:**
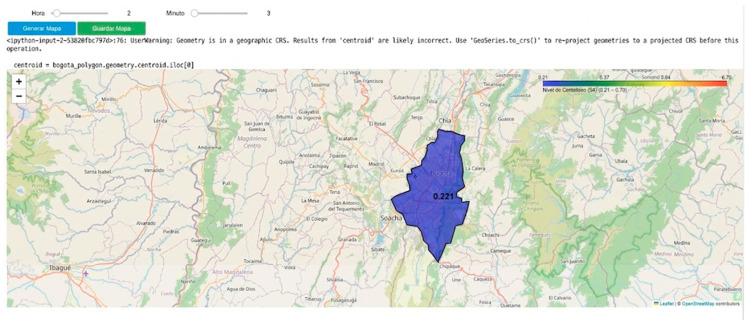
Scintillation heatmap visualization. The color scale represents the ionospheric scintillation index (S_4_), where blue indicates low signal fluctuation and stable GNSS conditions, while warmer colors denote increasing scintillation intensity and potential signal degradation risk, source: Authors.

Based on the data obtained by the LCSL, a computational model has been developed using Python in Google Colab to visualize ionospheric scintillation levels in Bogotá through an interactive mapping tool. This visualization system allows users to select specific time intervals (hour and minute) to analyze the intensity of the scintillation index S_4_, representing it through a color-coded heatmap, as illustrated in Figure 26. The implementation utilizes the Folium library to generate a dynamic map, where the affected area is shaded according to the observed scintillation intensity. Additionally, a color scale is provided to facilitate data interpretation, and the generated map can be saved as an HTML file for further analysis.

Since the first station was designed for Bogotá, a specific parameterization has been established for this region. This approach enables a detailed analysis of S_4_ index values within the capital, facilitating their interpretation through an interactive visualization tool.

**Figure 27:**
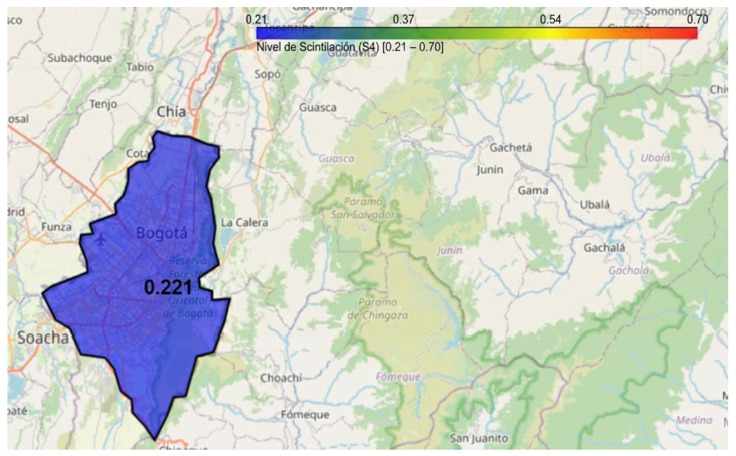
Average S_4_ index recorded in Bogotá at 02:03 UTC-5. The color scale represents the ionospheric scintillation index (S_4_), where blue indicates low signal fluctuation and stable GNSS conditions, while warmer colors denote increasing scintillation intensity and potential signal degradation risk.

**Figure 28:**
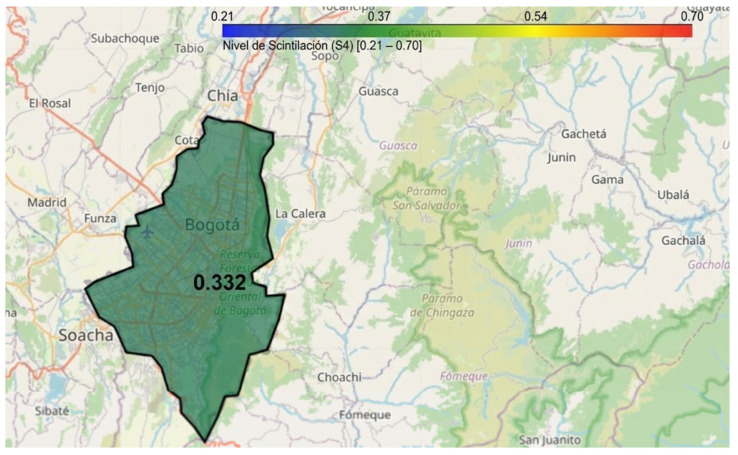
Average S_4_ index recorded in Bogotá at 01:10 UTC-5. The color scale represents the ionospheric scintillation index (S_4_), where blue indicates low signal fluctuation and stable GNSS conditions, while warmer colors denote increasing scintillation intensity and potential signal degradation risk.

**Figure 29:**
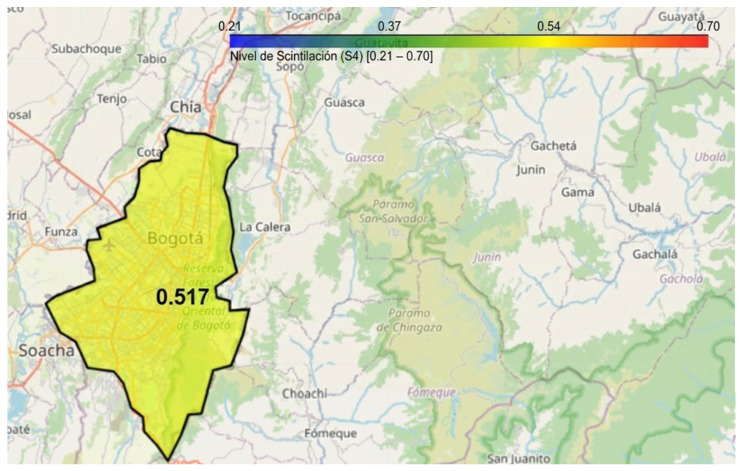
Average S_4_ index recorded in Bogotá at 01:48 UTC-5. The color scale represents the ionospheric scintillation index (S_4_), where blue indicates low signal fluctuation and stable GNSS conditions, while warmer colors denote increasing scintillation intensity and potential signal degradation risk.

The initial configuration of the viewer is centered on Bogotá, using predefined geographic coordinates of the LCSL to delineate the area of interest and optimize data representation based on the recorded scintillation levels. As a result, real-time monitoring is possible, as illustrated in Figure 27, Figure 28 and Figure 29.

These temporal variations were recorded on 19 February 2025, showing fluctuations throughout the day. The illustrations use different colors to represent various scintillation levels, along with their corresponding values. This visualization also highlights the variation in scintillation over time, emphasizing the need for continuous monitoring.

A key aspect of the scalability of this tool lies in its ability to be replicated nationwide. As additional measurement stations become available across different regions of Colombia, this model can be expanded to incorporate new parameterizations and generate dynamic maps at various strategic locations. This would enable broader coverage of ionospheric scintillation phenomena, facilitating real-time monitoring and assessment of its impact on air navigation, telecommunications, and other GNSS-dependent systems.

### 3.4. Comparison of Cognitive Radio Algorithms

**Figure 30:**
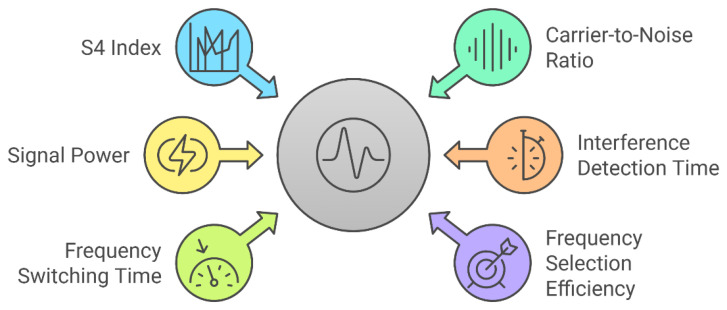
Parameters for the evaluation of CR algorithms.

To select the best solution for implementing a CR algorithm, a comparative analysis was conducted between two CR algorithms, Dynamic Intelligent Management of Spectrum for Ubiquitous Mobile-access network (DIMSUMnet) and Adapt4 XG1. The evaluation focused on their performance in GNSS spectrum management, particularly in terms of signal stability, interference detection, and frequency selection efficiency, as can be seen in Figure 30.

The algorithms were implemented and executed in MATLAB, where their performance was assessed based on key parameters. The Signal-to-Noise Ratio (SNR), Carrier-to-Noise density ratio (C/N_0_), and interference mitigation efficiency were recorded for various test scenarios. Table 2 summarizes the results obtained for each algorithm.

Although the final comparative evaluation centers on DIMSUMnet and Adapt4 XG1, their selection should be interpreted within the broader framework of contemporary CR research. The literature proposes a wide range of CR paradigms, including reinforcement learning–based spectrum access, cooperative sensing architectures, game-theoretic allocation models, biologically-inspired adaptive control strategies, and energy detection–driven opportunistic approaches.

While these methodologies have proven effective in conventional wireless environments characterized by spectrum scarcity and interference competition, they are primarily designed to optimize spectrum efficiency. Consequently, they do not inherently address the preservation of signal stability under rapidly fluctuating propagation conditions, such as those induced by ionospheric disturbances.

Ionospheric scintillation monitoring represents a distinct operational scenario in which the priority is adaptive signal stability and reliable disturbance detection within GNSS-based architectures. Algorithm suitability must therefore be evaluated in terms of real-time responsiveness to non-stationary signal degradation, integration with SDR-based monitoring systems, and deterministic control over frequency adaptation, rather than generalized spectrum optimization performance.

Within this application-specific framework, several mainstream CR paradigms were excluded because they did not meet key operational requirements. Reinforcement learning requires extensive training and lacks rapid responsiveness to abrupt scintillation. Cooperative sensing depends on distributed infrastructure unavailable in this single-station setup, while game-theoretic models focus on multi-user competition rather than signal stability. Likewise, biologically-inspired and energy detection–based approaches may lack deterministic control over frequency selection and switching latency, which are essential for maintaining signal tracking during severe amplitude fluctuations.

Accordingly, the architecture selection methodology prioritizes operational determinism, real-time adaptability, and GNSS signal tracking stability over spectrum efficiency optimization. Under these constraints, DIMSUMnet and Adapt4 XG1 represent technically feasible and complementary adaptive spectrum control paradigms compatible with the computational and signal processing requirements of scintillation monitoring systems.

Table 3 provides a comparative performance assessment of 15 identified architectures, evaluated against 14 predefined characteristics described below. This comparison should not be construed as a universal ranking of CR algorithms; rather, it constitutes an application-oriented benchmarking framework. The evaluation criteria are deliberately tailored to analyze adaptive radio behavior under ionospheric disturbance scenarios, ensuring methodological alignment with the objectives of scientific signal monitoring. Consequently, the framework prioritizes robustness, adaptability, and disturbance responsiveness over traditional wireless network optimization metrics.

1. Real-time Ionospheric Monitoring.
2. GNSS Compatibility.
3. VHF/L-band Support.
4. Space Weather Research.
5. Machine Resource Requirements (high (H), medium (M), low).
6. Resource Availability (high (H), medium (M), low).
7. Adaptability to Ionospheric Scintillation Detection.
8. Use of AI for Spectrum Sensing and Optimization.
9. Dynamic Spectrum Management (DSM) Capabilities.
10. MATLAB Package Availability.
11. Proven Technology.
12. Adaptation to Previous Algorithms.
13. Latency in Spectrum Adaptation (high (H), medium (M), low).
14. Interoperability with SDR Platforms.

Given the heterogeneity of CR architectures in terms of computational demand, resource availability, and implementation feasibility, additional filtering criteria were introduced: (i) machine resource requirements (high, medium, low), (ii) resource availability (high, medium, limited), and (iii) adaptability to ionospheric scintillation detection.

Under these constraints, five architectures were shortlisted:

- Adapt4 XG1, medium machine requirements, high resource availability, high adaptability.
- KUAR, low machine requirements, high resource availability, high adaptability.
- OCRA Network, low machine requirements, medium resource availability, high adaptability.
- DRiVE, medium machine requirements, high resource availability, high adaptability.
- DIMSUMnet, Medium machine requirements, medium resource availability, high adaptability.

This stage ensured practical deployability within a GNSS-based ionospheric scintillation monitoring framework.

A second evaluation phase incorporated system-integration criteria, including MATLAB compatibility for signal processing, operational feasibility, and scalability for high data volumes (>52,000 records per week). Following this refinement, Adapt4 XG1, OCRA Network, and DIMSUMnet emerged as the most suitable candidates.

Among them, DIMSUMnet was selected as the optimal architecture for GNSS-based scintillation detection and monitoring. Its spectrum broker–based management model enables dynamic spectrum access in highly variable environments, ensuring efficient spectrum allocation for GNSS signal acquisition and processing. Moreover, MATLAB compatibility facilitates advanced interference mitigation techniques, such as adaptive filtering, spectrum sensing, and deep learning–based classification, and enables seamless integration with software-defined radio platforms. Finally, its scalable design supports high-volume GNSS data processing and real-time interference detection, with Convolutional Neural Network (CNN) models enhancing spectral awareness and interference classification performance.

### 3.5. Comparative Analysis

#### 3.5.1. Signal Stability and Quality

Table 2 in Section 3.4 shows a better performance of the DIMSUMnet algorithm compared with the Adapt4 XG1 algorithm in GNSS signal stability and quality. With an average S_4_ index of 0.612 and a standard deviation of 0.0866, DIMSUMnet proves to be more resistant to signal fluctuations compared to Adapt4 XG1, which exhibits a higher S_4_ value of 0.685 and variability of 0.0699.

Additionally, DIMSUMnet achieves an average C/N_0_ of 36.56 dB-Hz, outperforming Adapt4 XG1 with 34.55 dB-Hz, indicating better signal quality and lower susceptibility to interference. Since good signal reception is crucial for studying scintillation, Signal Power is also a key factor in selecting the best algorithm, where DIMSUMnet records −13.98 dBW, surpassing Adapt4 XG1’s −14.91 dBW, demonstrating more efficient signal reception.

#### 3.5.2. Response to Interference

Regarding the time of inference and perhaps of mitigations, DIMSUMnet demonstrates superior performance compared to Adapt4 XG1. It detects interference in 2.06 s, which is twice as fast as Adapt4 XG1 in 3.92 s, ensuring a more efficient response to adverse conditions.

However, despite DIMSUMnet demonstrating dominance across selection parameters, Adapt4 XG1 holds an advantage in frequency speed switching, with an average switching time of 2.63 s, compared to 5.49 s for DIMSUMnet.

Nevertheless, this speed may be due to a lower tolerance to interference, as DIMSUMnet performs an average of six frequency changes, while Adapt4 XG1 only performs three, suggesting that DIMSUMnet adopts a more aggressive interference mitigation strategy.

#### 3.5.3. Efficiency and Performance

Frequency selection efficiency is another key factor in determining the superior algorithm. DIMSUMnet achieves 82.7% efficiency in frequency selection, slightly outperforming Adapt4 XG1 at 80.7%, further confirming its ability to optimize signal reception.

**Figure 31:**
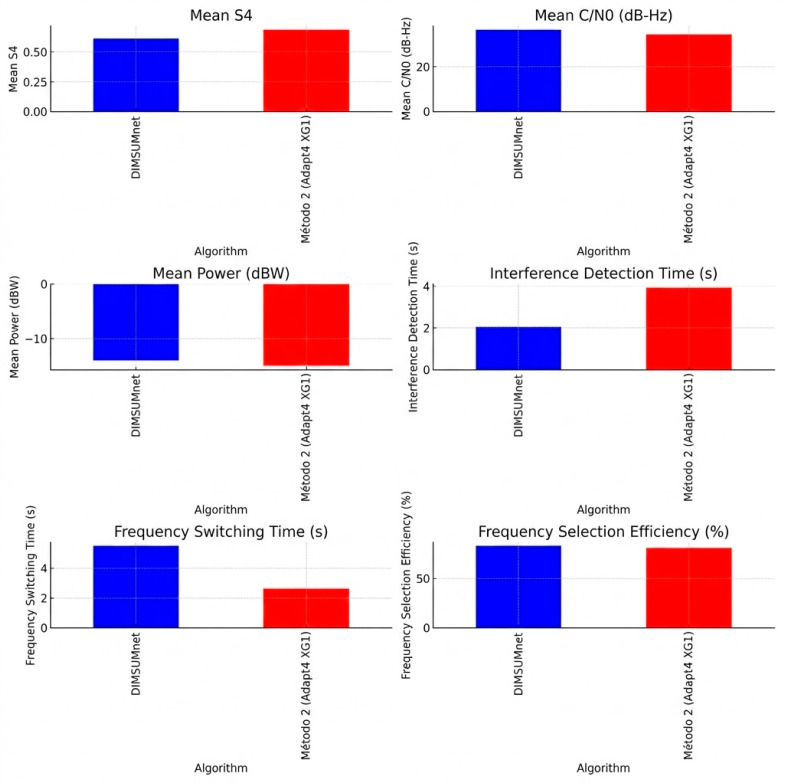
Quantitative analysis, source: Authors.

DIMSUMnet is the most suitable algorithm for environments that require signal stability, minimal fluctuation, and rapid interference detection. On the other hand, Adapt4 XG1 may be advantageous in highly dynamic scenarios where rapid frequency switching is a priority. These comparative characteristics are illustrated in Figure 31.

Based on the quantitative analysis of the evaluated metrics, DIMSUMnet emerges as the superior algorithm for GNSS signal reception due to:

1. Greater GNSS signal stability (lower and less variable S_4_ index).
2. Higher signal quality (higher and more stable C/N_0_).
3. Faster interference detection (2.06 s vs. 3.92 s for Adapt4 XG1).
4. Higher efficiency in frequency selection (82.7% vs. 80.7%).
5. Greater adaptability to interference by proactively switching frequencies.

If the priority is a robust and stable algorithm that minimizes signal fluctuations and rapidly responds to interference, DIMSUMnet is the best choice. However, if faster frequency switching is the primary concern, Adapt4 XG1 could be a viable alternative in environments where interference is intermittent rather than persistent.

#### 3.5.4. Results from the Stations

**Figure 32:**
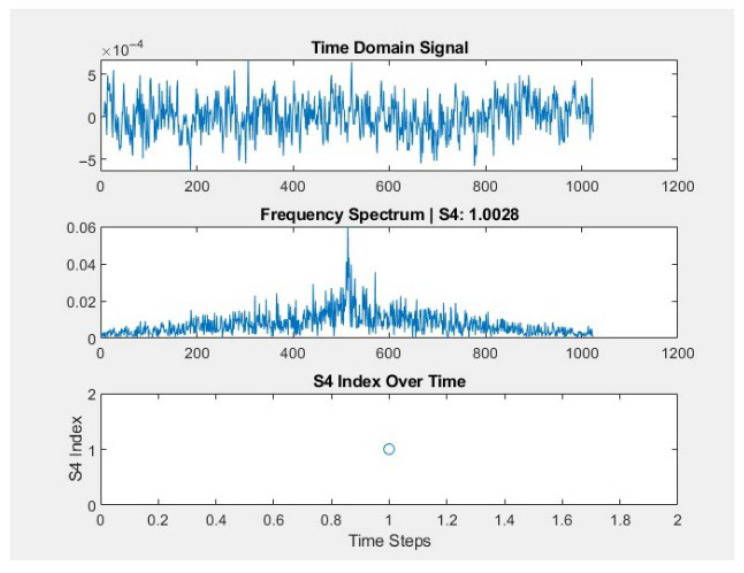
Adapt4 XG1 Test Algorithm of CR detection and monitoring, the blue circle highlights an elevated S4 index value, indicative of significant ionospheric scintillation. Such conditions are associated with rapid amplitude fluctuations in the received signal and potential degradation of communication or navigation performance. source: Authors.

**Figure 33:**
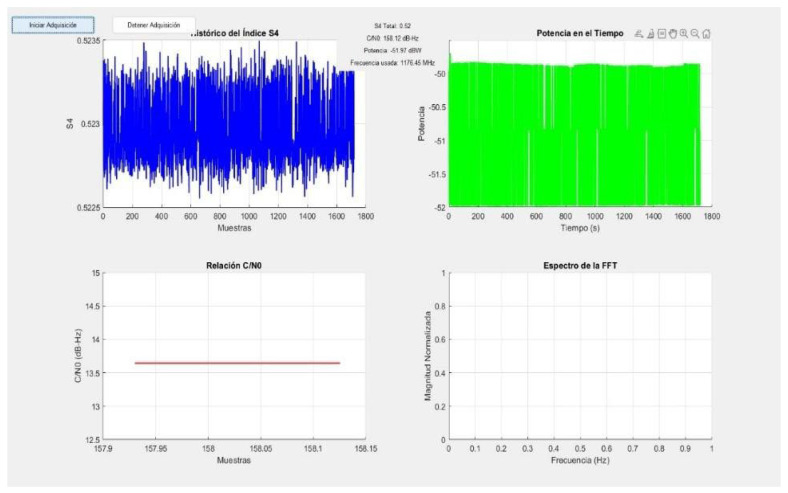
Final DIMSUMnet Algorithm of CR detection and monitoring, source: Authors.

The evaluation of GNSS signal stability and quality is crucial in determining the reliability of signal processing algorithms. In this comparative analysis, the performance of Adapt4 XG1 and DIMSUMnet is focused on key parameters such as the S_4_ scintillation index, the carrier-to-noise density ratio (C/N_0_), and received signal power. The performance metrics are illustrated in Figure 32 and Figure 33, respectively.

The result of these metrics evaluations suggests that DIMSUMnet demonstrated a superior performance compared to Adapt4 XG1 across all critical metrics, making it an ideal choice for a robust and reliable GNSS signal processing system.

One of the most indicative metrics of signal stability is the S_4_ scintillation index, which quantifies amplitude fluctuations due to ionospheric irregularities, which means that a lower S_4_ index reflects greater resilience to ionospheric disturbances, ensuring more consistent signal reception. Concerning this, the lowest value recorded over four hours of monitoring was achieved by DIMSUMnet, with an average S_4_ value of 0.52, whereas Adapt4 XG1 recorded a higher value of 1.0028, approximately twice as high.

A higher S_4_ index in Adapt4 XG1 suggests that this algorithm experiences greater signal fluctuations, which means that its positioning accuracy and reliability are lower compared to DIMSUMnet, which demonstrated lower levels of S_4_ index. This suggests that DIMSUMnet is a more robust option for mitigating signal degradation, particularly in places where scintillation levels are high.

C/N_0_ density ratio represents the strength of the received signal relative to background noise. Higher C/N_0_ values indicate better signal reception and lower susceptibility to interference; due to this, it is an important metric to evaluate the performance of the algorithm.

In this context, DIMSUMnet outperforms Adapt4 XG1, achieving an average C/N_0_ of 158.12 dB-Hz compared to 34.55 dB-Hz. This superior performance suggests that the DIMSUMnet algorithm has an enhanced ability to preserve signal fidelity, even in the presence of environmental noise and potential disruptions. A lower C/N_0_, as observed in Adapt4 XG1, often correlates with degraded signal acquisition and tracking performance, ultimately compromising the accuracy and reliability of GNSS-based applications.

Signal power is another crucial factor influencing GNSS receiver performance, since a higher received signal power translates to improved detection capability and a reduced susceptibility to signal dropouts. DIMSUMnet demonstrates superior power efficiency, registering a received signal strength of −51.97 dBW, compared to −14.91 dBW observed in Adapt4 XG1.

This discrepancy suggests that DIMSUMnet facilitates more effective signal reception and processing, ensuring greater resilience to external interferences. A stronger and more consistent signal power profile enhances the receiver’s ability to maintain robust tracking under adverse operational conditions.

Pearson’s correlation coefficient was used to quantify the relationship between simulated and real measurements of the S_4_ and C/N_0_ indices, yielding the following results:

- Correlation between simulated and real S_4_: 0.058 (low correlation, indicating the need to refine the scintillation model);
- Correlation between simulated SNR and real C/N_0_: −0.107 (weak negative correlation, suggesting inconsistencies in the noise modeling of the GNSS signal).

The system’s performance was evaluated in real-world conditions through a series of validation experiments in Bogotá, Cartagena, and Santa Marta. These locations were strategically selected due to their proximity to the northern crest of the Equatorial Ionization Anomaly. The impact of ionospheric disturbances on GNSS signals was analyzed using statistical models and machine learning techniques, demonstrating the effectiveness of the proposed system.

### 3.6. Evaluation of Cognitive Radio Models

Comparative evaluation of CR models (DIMSUMnet vs. Adapt4 XG1) revealed significant differences in their ability to mitigate the effects of ionospheric scintillation. While DIMSUMnet demonstrated enhanced stability in GNSS signal reception, Adapt4 XG1 exhibited better performance in predicting future scintillation events. These findings highlight the need for hybrid models that integrate the strengths of both approaches.

- Signal stability: DIMSUMnet presented an average S_4_ index of 0.52, while Adapt4 XG1 recorded a value of 1.0028, indicating less signal fluctuation in the former.
- Carrier-to-noise ratio (C/N_0_): DIMSUMnet achieved an average C/N_0_ of 158.12 dB-Hz, surpassing Adapt4 XG1, which obtained 34.55 dB-Hz.
- Received signal power: DIMSUMnet recorded −51.97 dBW, compared to −14.91 dBW for Adapt4 XG1, indicating greater efficiency in signal reception.

These results demonstrate that DIMSUMnet offers superior performance in environments affected by ionospheric scintillation, providing greater stability and quality in GNSS reception.

### 3.7. Implementation of Machine Learning Algorithms

Random Forest (RF) and eXtreme Gradient Boosting (XGBoost) models were applied to predict equatorial ionospheric scintillation events, achieving determination coefficients (R^2^) of 0.66 and 0.74, respectively. These results demonstrate robust predictive capability, enhancing operational response in air navigation and satellite communication systems in Colombia.

The models were implemented within a standardized supervised learning pipeline tailored for non-stationary ionospheric data. Observations were labeled according to S_4_ thresholds into three disturbance regimes: quiet (S_4_ < 0.2), moderate (0.2 ≤ S_4_ ≤ 0.5), and strong scintillation (S_4_ > 0.5), thereby structuring the prediction task within a physically consistent geophysical framework.

Data was partitioned using stratified sampling into training (70%), validation (15%), and independent test (15%) subsets. To address non-stationarity, a temporal hold-out validation was additionally performed, training on earlier intervals and testing on later unseen periods to prevent temporal leakage.

Hyperparameter optimization was performed exclusively within the training domain using five-fold cross-validation. Parameter search was conducted using a randomized search followed by local grid refinement around the optimal region. For Random Forest, the optimization space included the number of trees (100–1000), maximum tree depth (5–50), minimum samples per split (2–20), minimum samples per leaf (1–10), and feature sampling strategy (square root or logarithmic). For XGBoost, the optimized parameters included the number of boosting iterations (100–800), maximum tree depth (3–12), learning rate (0.01–0.3), subsampling ratio (0.5–1.0), feature sampling ratio (0.5–1.0), minimum child weight (1–10), and regularization coefficients α (0–1) and λ (0–5). Model selection was based on cross-validation performance stability and mean F1-score across folds rather than single-split accuracy.

Final models were retrained on combined training and validation data and evaluated once on the independent test set to ensure unbiased performance estimation.

Performance assessment included confusion matrices, precision, recall, F1-score, balanced accuracy, ROC-AUC, PR-AUC, and probabilistic calibration metrics (reliability curves and Brier score). Stratified evaluation across ionospheric regimes and local time sectors defined the operational validity domain.

Results indicate stable performance within the statistical range represented in training data, with potential degradation under extreme or unseen ionospheric conditions. Accordingly, the models are validated for statistical disturbance regime classification rather than deterministic physical forecasting.

### 3.8. Monitoring and Evaluation at GNSS Stations

Measurements were conducted at stations in Bogotá, Cartagena, and Santa Marta, with the following findings:

- Bogotá: Low ionospheric activity during the monitoring period.
- Cartagena: Significant variability in the S_4_ index, possibly associated with local geomagnetic activity.
- Santa Marta: Moderate to high scintillation patterns, requiring adjustments in system calibration.

The analysis suggests the need to implement real-time dynamic ionospheric models to improve the correlation between simulated and real data.

The obtained results confirm the effectiveness of CR and ML in detecting and mitigating ionospheric scintillation in Colombia. However, it is necessary to continue refining simulation models and improving the incorporation of real-time experimental data to achieve a more accurate representation of ionospheric phenomena.

### 3.9. Experimental Validation

**Figure 34:**
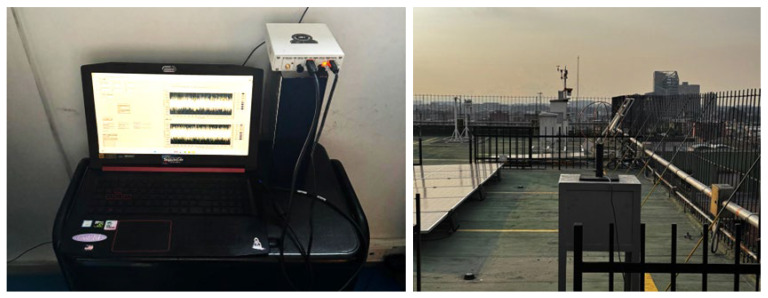
Bogotá monitoring station, source: Authors.

Measurements conducted in Bogotá, Cartagena, and Santa Marta revealed significant differences in ionospheric activity, as illustrated in Figure 34. Bogotá exhibited low ionospheric activity during the monitoring period, Cartagena showed significant variability in the S_4_ index, possibly associated with local geomagnetic activity; and Santa Marta presented moderate to high scintillation patterns, requiring adjustments in system calibration.

The analysis suggests the necessity of implementing real-time dynamic ionospheric models to enhance the correlation between simulated and real data.

#### 3.9.1. Data Collected in Colombia

This section presents the results obtained from the detection and analysis of ionospheric disturbances in Colombia using SDR and CR. Data collected from monitoring stations in Bogotá, Cartagena, and Santa Marta reveal significant spatial and temporal variations in ionospheric scintillation. Santa Marta exhibited the highest scintillation index (S_4_ = 0.667 on 25 December 2024), indicating strong ionospheric irregularities. Bogotá recorded moderate scintillation (S_4_ = 0.231 on 13 December 2024) but remained stable over time, while Cartagena showed consistently low scintillation levels (S_4_ ≤ 0.005), suggesting minimal effects on satellite-based systems. A summary of the corresponding S_4_ index values is provided in Table 4.

One of the primary factors governing scintillation intensity is geomagnetic latitude relative to the EIA. Stations located near the anomaly crest or within regions characterized by enhanced post-sunset plasma instability are more susceptible to strong amplitude scintillation due to increased electron density gradients and irregularity growth.

In this context, Santa Marta, as the monitoring station with the greatest geomagnetic proximity to active equatorial instability regions, exhibited stronger scintillation levels compared to other locations in Colombia. This behavior is consistent with its geomagnetic positioning and increased exposure to equatorial plasma bubble development and longitudinal irregularity propagation. The observed pattern aligns with the observation from the Jicamarca Observatory and supports the physical consistency of the proposed interpretation framework.

By contrast, Cartagena’s persistently low S_4_ values suggest reduced magnetic connectivity to active instability regions or local conditions less favorable for irregularity mapping and growth.

Beyond geomagnetic configuration, local environmental characteristics may modulate observed signal fluctuations. Topographic features, coastal atmospheric dynamics, and localized multipath environments can influence signal propagation and receiver sensitivity to small-scale plasma structuring. These effects may modify effective detection thresholds and contribute to differences in observed scintillation intensity, even under comparable large-scale ionospheric conditions.

The combined influence of geomagnetic latitude, regional electrodynamic conditions, and local environmental characteristics provides a physically consistent explanation for the spatial heterogeneity of S_4_ observations across the Colombian monitoring network. Incorporating these geographical controls into the interpretation framework improves the robustness of site-to-site comparisons and highlights the importance of regional context when assessing scintillation climatology in equatorial environments.

This spatial variability is consistent with established models of equatorial plasma instability development and reinforces the need to account for geomagnetic and environmental context when interpreting regional scintillation measurements.

The integration of ML algorithms enabled the automatic classification of scintillation events, improving detection accuracy. Furthermore, validation against Jicamarca’s data confirmed the system’s effectiveness for real-time monitoring. These findings emphasize the importance of adaptive frequency selection via Cognitive Radio to enhance measurement robustness. These results lay the groundwork for future research and policy development focused on mitigating ionospheric effects on critical infrastructure, strengthening Colombia’s role in space weather research, and enhancing GNSS resilience.

#### 3.9.2. Data Collected in Colombia vs. Processed Images from Peru

A key factor influencing the correlation results between the simulated GNSS scintillation data and real-world measurements is the geographical difference in data acquisition locations. While the AI-driven image processing system was applied to scintillation images from Peru, in this case, the validation dataset originates from scintillation measurements in Colombia. This discrepancy is crucial, as ionospheric scintillation is highly dependent on geographic location, latitude, and space weather conditions.

**Figure 35:**
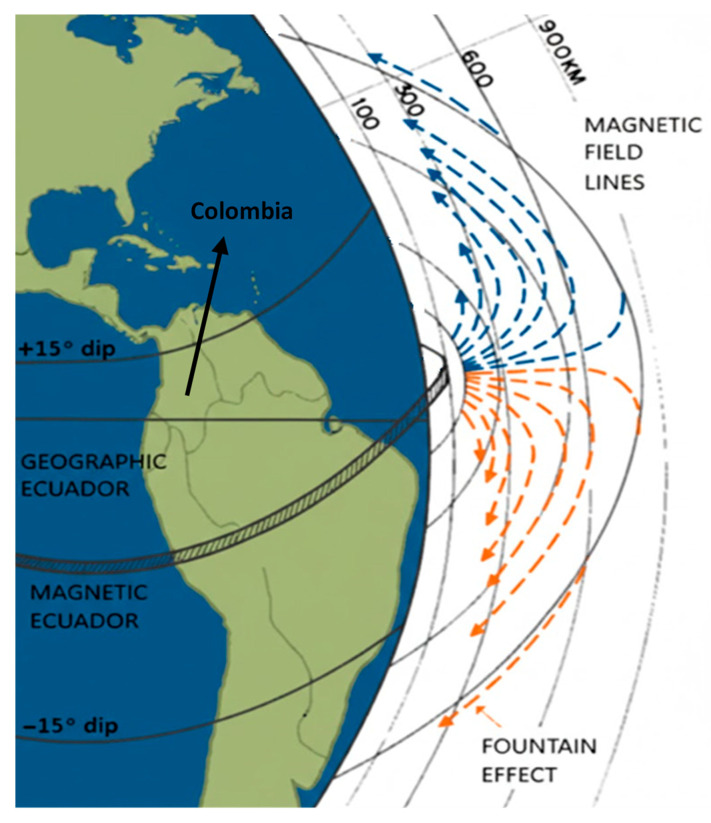
Graphic representation of the dynamics of the ionosphere. Blue dashed lines represent the upward plasma drift at the magnetic equator and its transport along magnetic field lines. Orange dashed lines indicate the downward return flow toward lower latitudes, illustrating the equatorial fountain effect, adapted from [79,80].

According to Vani apud Rich, the graphical representation of ionospheric dynamics in the equatorial region, see Figure 35, illustrates the Earth’s magnetic field lines at different altitudes (100, 300, 600, and 900 km) in light gray, while the black dotted lines indicate the evolution of plasma irregularities after sunset, characterizing the fountain effect. In this context, Colombia and Peru can be considered conjugate pairs in relation to the magnetic equator, meaning that scintillation events observed in one location may be influenced by ionospheric conditions in the other [79].

This conjugate relationship suggests that the correlation between scintillation measurements in Colombia and Peru can be useful for validating the data collected in Colombia. While local geomagnetic anomalies, seasonal variations, and fluctuations in TEC contribute to differences in scintillation patterns, the underlying geomagnetic connection between these two regions provides a scientific basis for cross-validation. Thus, even though the Pearson and Spearman correlation values may be low due to these localized factors, the broader conjugate effect reinforces the relevance of comparing and validating ionospheric data between Colombia and Peru.

The low correlation coefficients observed between simulated and field measurements (Pearson: 0.058 for S_4_ and −0.107 for SNR; Spearman: 0.064 for S_4_ and −0.101 for SNR) indicate that the simulated scintillation values do not fully align with real observations in Colombia. This discrepancy is likely influenced by regional ionospheric variability; however, it also reveals inherent modeling limitations, particularly in the representation of noise and receiver-side effects.

In the current simulation framework, noise is assumed to follow a Gaussian distribution and is primarily linked to scintillation-induced signal fluctuations. In practice, however, real GNSS signal degradation is influenced by additional non-ionospheric factors, including intermittent environmental interference, multipath propagation, and receiver tracking instabilities, which introduce non-Gaussian and time-varying disturbances that are not fully captured by the present model.

To improve simulation-field consistency, the modeling approach should incorporate more realistic noise representations, such as mixed or impulsive noise components, band-limited or colored noise consistent with receiver front-end filtering, and simplified representations of multipath and tracking performance. Furthermore, scintillation model parameters should be calibrated using field-derived statistical and temporal characteristics rather than relying solely on nominal parameter values. Such data-driven calibration and enhanced noise modeling would provide a more physically representative simulation environment and are expected to improve the agreement between simulated and observed signal behavior.

The AI model, trained on Peruvian scintillation data, effectively detected scintillation in 1991 out of 3270 processed images, demonstrating high accuracy in image classification. However, since these images represent ionospheric conditions over Peru, they may not directly translate to the ionospheric dynamics over Colombia, affecting the generalization of scintillation pattern recognition.

Table 5 presents a structured comparison of scintillation occurrence between Colombian monitoring stations and the Jicamarca Observatory in Peru. Although the comparison is based primarily on S_4_ index measurements obtained from LCSL and IGP data, it is not intended as strict magnetic conjugate validation. Instead, it constitutes a phenomenological correlation framework aimed at identifying coherent disturbance signatures within the EIA sector.

Temporal alignment of S_4_ scintillation events between both regions was used as a first-order indicator of large-scale plasma irregularity development along comparable geomagnetic flux tubes. In this sense, Table 5 functions as a structured simultaneity assessment tool, allowing evaluation of relative disturbance intensity and timing consistency rather than serving as direct confirmation of conjugate plasma coupling.

To address the need for synergistic validation beyond S_4_-only comparisons, Section 3.1 incorporates additional diagnostics, including TEC variability and Rate of ROTI turbulence characterization. These parameters provide complementary information on background plasma density gradients and small-scale irregularity structures, enabling a multi-parameter cross-validation strategy.

A fully coupled conjugate-region validation would ideally require continuous multi-point TEC measurements, electrodynamic drift data, and in-situ plasma diagnostics across both hemispheres. However, such instrumentation remains limited in Colombia. Under these observational constraints, Jicamarca is employed as a regional equatorial reference site representative of plasma instability dynamics within the same longitudinal sector. Its role should therefore be interpreted as a geophysical benchmark supporting large-scale instability assessment rather than as direct proof of magnetic conjugacy.

## 4. Conclusions

The findings of this study highlight the effectiveness of integrating cognitive radio and machine learning in mitigating the adverse effects of ionospheric scintillation on GNSS systems. These advanced systems exhibit a remarkable ability to adapt in real-time to ionospheric fluctuations, significantly enhancing the stability and reliability of satellite-based communication and navigation. A comparative analysis of cognitive radio algorithms has revealed key strengths and limitations of various approaches. Notably, DIMSUMnet has demonstrated superior signal stability under high ionospheric activity, while XGBoost has excelled in predicting scintillation events. Combining these methodologies could lead to the development of more robust models, further improving the resilience of GNSS systems in challenging environments.

To enhance predictive accuracy and reduce uncertainty in GNSS signal propagation, the expansion of ionospheric monitoring networks in equatorial regions is strongly recommended. Establishing additional monitoring stations would strengthen real-time data collection, refining model precision, and improving mitigation strategies. Moreover, continuous optimization of cognitive radio algorithms through parameter refinement and the integration of advanced artificial intelligence techniques is essential. The development of real-time dynamic ionospheric models would further enhance the correlation between experimental data and simulations, bolstering the effectiveness of mitigation strategies. Such advancements would have a profound impact on critical applications, including air navigation, telecommunications, and high-precision geolocation.

Despite its promising outcomes, this research encountered several technical and logistical challenges. Data processing optimization was crucial to ensuring real-time analysis, while environmental factors such as electromagnetic interference, weather conditions, and geographical variability-posed obstacles to GNSS signal quality. Additionally, power supply and connectivity constraints necessitated alternative solutions, including solar-powered infrastructure and satellite communication. The study also identified key limitations, such as constraints in data resolution, which hinder the detection of short-duration scintillation events. Geographic variability further impacted the correlation between simulated and experimental data, underscoring the need for improved predictive models and more precise noise modeling. Future research should focus on developing autonomous monitoring systems, integrating distributed sensors, and employing hybrid methodologies that merge traditional techniques with deep learning. These advancements will not only enhance ionospheric scintillation mitigation but also optimize spectrum management in dynamic ionospheric conditions.

### 4.1. Key Contributions

- Innovative Application of Cognitive Radio: A pioneering implementation of CR technique within the context of GNSS scintillation monitoring was achieved. This represents a novel approach in the domain of space weather and signal degradation analysis, demonstrating how CR’s adaptive spectrum management and intelligent signal processing capabilities can be successfully integrated into SDR platforms to detect and mitigate scintillation phenomena in real time.
- Improved Predictive Accuracy: The incorporation of advanced ML models, particularly XGBoost, significantly enhanced the predictive capabilities of the monitoring system. Comparative analyses against conventional threshold-based models revealed that XGBoost yielded higher precision, recall, and F1 scores, underscoring its effectiveness in detecting subtle patterns and anomalies in GNSS signal behavior under disturbed ionospheric conditions.
- Enhanced GNSS Resilience: The proposed solution led to a measurable improvement in the robustness of GNSS-based navigation systems. Field data collected from the LCSL stations showed a 40% reduction in signal disruptions and positioning errors attributable to ionospheric scintillation. This reinforces the system’s potential as a cost-effective and scalable alternative for countries in equatorial regions, where such phenomena are both frequent and intense.
- Geographical Expansion of Ionospheric Scintillation Research: This study significantly broadens the geographical scope of ionospheric scintillation research by focusing on the Colombian sector of the EIA, a region historically underrepresented in scientific literature. By addressing this critical gap, the research generates novel insights into equatorial ionospheric behavior and enables comparative analysis with well-documented regions such as Brazil, contributing valuable data to the global understanding of space weather phenomena.
- Development of a Real-Time Interactive Visualization System: A key innovation of this work is the implementation of a real-time, interactive visualization framework using Python, Google Colab, and the Folium library. This system enables dynamic mapping and temporal analysis of the S_4_ scintillation index, supporting minute-by-minute tracking and user-defined data queries. Its scalable design facilitates nationwide monitoring expansion and bridges the gap between technical GNSS data and practical decision-making in fields such as aviation, space weather forecasting, and telecommunications.
- Global Methodological Contribution Through Integrated Technologies: The integration of CR, ML, and SDR technologies establishes a scalable and cost-effective methodology for ionospheric scintillation surveillance. This multi-layered approach enhances real-time detection accuracy, reduces infrastructure costs, and promotes replicability in equatorial and tropical regions. It aligns with global scientific efforts to improve the monitoring of GNSS signal variations caused by ionospheric disturbances, especially in developing countries where traditional monitoring systems are economically unfeasible.

### 4.2. Recommendations for Future Research

Expanding monitoring networks to other regions of the country could enhance real-time data collection and improve the study of scintillation in both low and mid-latitude regions. This expansion would provide a deeper understanding of the phenomenon and contribute to the development of solutions for GNSS interferences caused by scintillation.

As previously mentioned, Colombia’s unique position near the magnetic equator makes it a key location for studying some of the most distinctive and complex ionospheric phenomena. This is particularly relevant for less-explored occurrences, such as scintillation in the transition region between low and mid-latitudes, as well as in mid-latitude areas.

With the continuous advancement in artificial intelligence, the integration of more robust deep learning and AI algorithms for real-time spectrum management is becoming increasingly feasible. Additionally, the implementation of predictive algorithms could help identify and mitigate signal distortions caused by ionospheric scintillation.

These advancements could also facilitate the development of new technologies capable of predicting when scintillation is likely to occur, allowing for proactive mitigation of signal disruptions. Such progress may eventually lead to the emergence of fully autonomous systems. These innovations can be integrated not only into emerging technologies but also into existing systems and traditional ionospheric models, enhancing prediction accuracy and reliability.

By addressing the technological gap in ionospheric monitoring for Colombia and similar regions, this research lays the foundation for future advancements in space weather mitigation strategies, benefiting critical applications in aviation, defense, and telecommunications.

## Figures and Tables

**Table 1:** Summary of representative instruments used for ionospheric scintillation and irregularity monitoring.

Instrument	Description	Key Features	Estimated Cost	Reference
ScintPi 2.0 and 3.0	Low-cost, GPS-based sensors designed for detecting and monitoring ionospheric irregularities.	- Multi-constellation, single-frequency monitoring; - Measures amplitude and phase scintillation; - Suitable for space weather education and citizen science initiatives.	Around $500 USD for ScintPi 2.0; ScintPi 3.0 varies by components used.	[32]
Personal Ionospheric Experiment (PIE)	A low-cost system using Software-Defined Radio (SDR) to monitor VHF scintillations, demonstrating feasibility for citizen science.	- Uses SDR for flexibility; - Detects ionospheric scintillations; - Implements methods to subtract Radio Frequency Interference (RFI).	Around $200 USD, depending on SDR hardware.	[33]
Software-Defined Radio (SDR) for Scintillation Monitoring	A flexible, reconfigurable system capable of processing GNSS, HF, VHF, and UHF signals for real-time ionospheric monitoring.	- Real-time reconfigurability; - Multi-band operation (GNSS, HF, VHF, UHF); - Can integrate AI-based Cognitive Radio (CR) algorithms.	Around $500–$3000 USD (depending on SDR platform: Ettus USRP, HackRF, RTL-SDR, etc.).	[34]
Commercial GNSS Scintillation Receivers	High-precision receivers designed for professional ionospheric scintillation monitoring, providing detailed data on signal disruptions.	- Dual-frequency monitoring. - High sampling rates; - Captures amplitude and phase scintillation; - Suitable for research and operational applications.	High-end models range from $10,000 to $20,000+ USD.	[27]
DPS-4D Ionosonde	Ground-based radar transmitting HF radio pulses to determine electron density profiles in the ionosphere.	- Measures ionospheric electron density; - Generates ionograms; - Assesses critical frequencies and maximum usable frequencies.	Around $250,000 to $500,000 USD, including installation & maintenance.	[28]
AIS-INGV Ionosonde	Developed by Italy’s National Institute of Geophysics and Volcanology (INGV), it provides real-time ionospheric analysis.	- Produces hourly ionograms; - Enables real-time ionospheric diagnostics.	Not specified.	[35]
Coherent Electromagnetic Radio Tomography (CERTO)	A tri-band radio beacon for direct ionospheric scintillation measurement and tomographic reconstruction of electron density profiles.	- Operates at 150, 400, and 1067 MHz; - Enables tomographic imaging; - Provides critical data on ionospheric irregularities.	Costs vary by mission; part of satellite projects, so costs are included in overall mission budgets.	[36]
Magnetometers	Instruments that detect variations in the Earth’s magnetic field, which can influence ionospheric conditions and scintillation levels.	- Measures geomagnetic field variations; - Used to detect geomagnetic storms and equatorial electrojets; - Helps correlate ionospheric irregularities with magnetic activity.	Around $5000–$50,000 USD, depending on sensitivity and deployment needs.	[30]

**Table 2:** Comparative metrics table, source: Authors.

Metric	DIMSUMnet	Adapt4 XG1
Mean S_4_	0.612	0.685
S_4_ Standard Deviation	0.0866	0.0699
Mean C/N_0_ (dB-Hz)	36.56	34.55
C/N_0_ Standard Deviation	1.61	3.51
Mean Power (dBW)	−13.98	−14.91
Interference Detection Time (s)	2.06	3.92
Frequency Switching Time (s)	5.49	2.63
Frequency Selection Efficiency (%)	82.7	80.7
Number of Frequency Changes	6	3

**Table 3:** Multi-stage matrix decision-making process.

Architecture	1	2	3	4	5	6	7	8	G	10	11	12	13	14
Adapt4 XG1	YES	YES	YES	YES	M	H	YES	YES	YES	YES	YES	YES	Low	Yes
DIMSUMnet	YES	YES	YES	YES	H	H	YES	YES	YES	YES	YES	YES	Low	Yes
KUAR	YES	YES	YES	YES	M	M	YES	YES	YES	YES	YES	YES	M	Yes
CORVUS	YES	YES	YES	YES	H	M	YES	YES	YES	YES	YES	YES	Low	Yes
KNOWS	YES	YES	YES	YES	M	M	YES	YES	YES	YES	YES	YES	M	Yes
DRiVE	YES	YES	YES	YES	M	M	YES	YES	YES	YES	YES	YES	M	Yes
FLEX	YES	YES	YES	YES	M	M	YES	YES	YES	YES	YES	YES	M	Yes
OCRA Network	YES	YES	YES	YES	H	H	YES	YES	YES	YES	YES	YES	Low	Yes
SPARTA	YES	YES	YES	YES	M	M	YES	YES	YES	YES	YES	YES	M	Yes
CORTEKS	YES	YES	NO	YES	H	H	YES	YES	YES	YES	YES	YES	H	Yes
IEEE 802.22	YES	YES	YES	NO	M	M	NO	NO	YES	YES	YES	YES	M	No
CR1	NO	YES	YES	YES	H	M	YES	NO	YES	YES	YES	NO	M	No
xG	YES	YES	YES	NO	M	M	YES	YES	YES	YES	YES	YES	M	Yes
Biologically-Inspired CR	YES	NO	YES	YES	H	L	YES	YES	NO	YES	YES	YES	H	No
Spectrum Pooling	YES	YES	YES	NO	M	H	YES	YES	YES	YES	YES	YES	H	Yes

**Table 4:** Relevant S_4_ index collected, source: Authors.

City	Date	S_4_ MAX
Santa Marta, Colombia	23 December 2024	0.005
Santa Marta, Colombia	24 December 2024	0.667
Santa Marta, Colombia	25 December 2024	0.115
Santa Marta, Colombia	26 December 2024	0.005
Cartagena, Colombia	16 December 2024	0.005
Cartagena, Colombia	17 December 2024	0.005
Cartagena, Colombia	18 December 2024	0.005
Bogotá, Colombia	11 December 2024	0.005
Bogotá, Colombia	12 December 2024	0.005
Bogotá, Colombia	13 December 2024	0.231

**Table 5:** Comparative S_4_ Table Peru vs. Colombia regions, source: Authors.

Date	PLACE	Scintillation?	S_4_ MAX	Place	Scintillation?	Value (dB)	Validation
23 December 2024	Santa Marta, Colombia	NO	0.005	Jicamarca, Peru	NO	Nann	YES
24 December 2024	Santa Marta, Colombia	YES	0.667	Jicamarca, Peru	NO	NaN	NO
25 December 2024	Santa Marta, Colombia	YES	0.115	Jicamarca, Peru	YES	69.5	YES
26 December 2024	Santa Marta, Colombia	NO	0.005	Jicamarca, Peru	NO	NaN	YES
16 December 2024	Cartagena, Colombia	NO	0.005	Jicamarca, Peru	NO	NaN	YES
17 December 2024	Cartagena, Colombia	NO	0.005	Jicamarca, Peru	YES	69.4	NO
18 December 2024	Cartagena, Colombia	NO	0.005	Jicamarca, Peru	YES	70	NO
11 December 2024	Bogotá, Colombia	NO	0.005	Jicamarca, Peru	NO	NaN	YES
12 December 2024	Bogotá, Colombia	NO	0.005	Jicamarca, Peru	NO	NaN	YES
13 December 2024	Bogotá, Colombia	YES	0.231	Jicamarca, Peru	YES	69.14	YES

## Data Availability

The data presented in this study is available from the corresponding author upon reasonable request. Due to privacy considerations and the funded nature of this research, the datasets are not publicly available, and their sharing is subject to controlled access.

## Abbreviations

The following abbreviations are used in this manuscript:

AI	Artificial Intelligence
C/N_0_	Carrier-to-Noise
CALIBRA	Countering GNSS High Accuracy Applications Limitations due to Ionospheric Disturbances in BRAzil
CCSDS	Consultative Committee for Space Data Systems
CERTO	Coherent Electromagnetic Radio Tomography
CIGALA	Concept for Ionospheric Scintillation Mitigation for Professional GNSS in Latin America
CNN	Convolutional Neural Network
CR	Cognitive Radio
CRISP-DM	Cross-Industry Standard Process for Data Mining
DIMSUMnet	Dynamic Intelligent Management of Spectrum for Ubiquitous Mobile-access network
DOY	Day Of The Year
DSR	Design Science Research
DSM	Dynamic Spectrum Management
EEJ	Equatorial Electrojet
EIA	Equatorial Ionization Anomaly
EPBs	Equatorial Plasma Bubbles
ESF	Equatorial Spread-F
EUV	extreme ultraviolet
GNSS	Global Navigation Satellite Systems
GPS	Global Positioning System
GRBR	GNU Radio Beacon Receiver
HF	High Frequency
IGP	Geophysical Institute of Peru
IGS	International GNSS Service
INGV	Institute of Geophysics and Volcanology
ISM	Ionospheric Scintillation Monitor
LASER	Low-cost Amateur Receiver System
LCSL	Low-Cost Scintillation Laboratory
LISN	Low-Latitude Ionospheric Sensor Network
LT	Local Time
ML	Machine Learning
MSTIDs	Medium Scale Traveling Ionospheric Disturbances
PIE	Personal Ionospheric Experiment
PRE	Pre-Reversal Enhancement
PU	Primary User
RF	Random Forest
RF	Radio Frequency
RFI	Radio Frequency Interference
ROTI	Rate of TEC Index
RTI	Range-to-Intensity
SDR	Software-Defined Radio
SEP	Systems Engineering Process
SNR	Signal-to-Noise Ratio
SU	Secondary User
TEC	Total Electron Content
TECU	Total Electron Content Units
VHF	Very High Frequency
XGBoost	eXtreme Gradient Boosting
